# Supplementary material for: 2-Arylpropionic Acid Pyrazolamides as Cannabinoid CB2 Receptor Inverse Agonists Endowed with Anti-Inflammatory Properties
Source: Pharmaceuticals (Basel). 2022 Dec 6;15(12):1519. doi: 10.3390/ph15121519 (PMC9781268; doi:10.3390/ph15121519)
Supplement: Supplementary file 1 [file pharmaceuticals-15-01519-s001.zip › pharmaceuticals-1977773-supplementary.pdf]

# SUPPORTING INFORMATION

## 2-Arylpropionic Acid Pyrazolamides as Cannabinoid CB2 Receptor Inverse Agonists Endowed with Anti-inflammatory Properties

**Daniela R. de Oliveira [2], Rodolfo C. Maia [1], Patrícia R. de Carvalho [2,3], Patrícia D. Fernandes [2,3], Gisele Barbosa [1,2], Lídia M. Lima [1,2] and Carlos A. Manssour Fraga [1,2,*]**

1 Laboratório de Avaliação e Síntese de Substâncias Bioativas, Instituto de Ciências Biomédicas, Universidade Federal do Rio de Janeiro, Rio de Janeiro, RJ, 21920-190, Brazil
2 Programa de Pós-Graduação em Farmacologia e Química Medicinal, Instituto de Ciências Biomédicas, Universidade Federal do Rio de Janeiro, Rio de Janeiro, RJ, 21920-190, Brazil
3 Laboratório da Dor e Inflamação, Instituto de Ciências Biomédicas, Universidade Federal do Rio de Janeiro, Rio de Janeiro, RJ, 21920-190, Brazil
* Correspondence: cmfraga@ccsdecania.ufrj.br

CONTENT:

**FIGURES S1-S6**: Structural characterization of pyrazolamide (**7**);
**FIGURES S7-S12**: Structural characterization of pyrazolamide (**8**);
**FIGURES S13-S18**: Structural characterization of pyrazolamide (**9**);
**FIGURES S19-S23**: Structural characterization of pyrazolamide (**10**);
**FIGURES S24-S28**: Structural characterization of pyrazolamide (**11**);
**FIGURES S29-S30**: Structural characterization of bismethylated pyrazolamide (**16**);
**FIGURES S31-S32**: Alternative methodology tried for the synthesis pyrazolamide (**12**);
**FIGURE S33**: Calibration curves for determination of aqueous solubility of the pyrazolamides (**7-11**);
**FIGURE S34**: pH-Dependent chemical stability of pyrazolamides (**7-11**);
**FIGURES S35-S36**: Absorbed fraction of drugs used as standard of GIT and BBB-PAMPA model and pyrazolamides (**7-11**);
**FIGURES S35-39**: Plasma stability profile of pyrazolamides (**7-11**);
**Figure S40.** Dose-response curve of inverse agonism for cannabinoid receptor 2 (CB2) performed by the company Eurofins-CEREP: (**A**) control inverse agonist SR 144528 and (**B**) LASSBio-2265 (**11**).

**Table S1.** Absorbed fraction of drugs used as standard and pyrazolamides **(7-11)** determined in the PAMPA-GTI model.
**Table S2.** Permeability of drugs used as standard, and pyrazolamides **(7-11)** determined in the PAMPA-BBB model.
**Table S3.** Evaluation the agonist effect against the CB1 receptors of the pyrazolamides (**7-11**).

**Table S4.** Evaluation the agonist effect against the CB2 receptors of the pyrazolamides (**7-11**).

**Table S5.** Raw data from the inverse agonism assay performed by the company Eurofins-CEREP

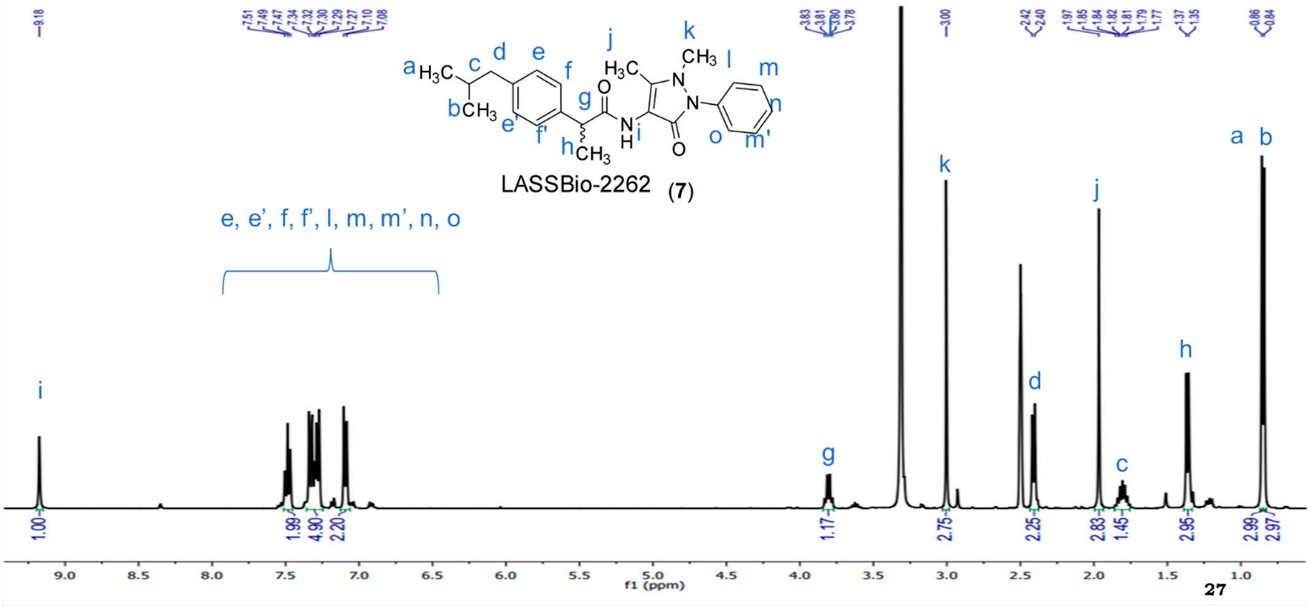

**Figure S1.** NMR spectrum¹H pyrazolamide **(7)** (400 MHz, DMSO-d6).

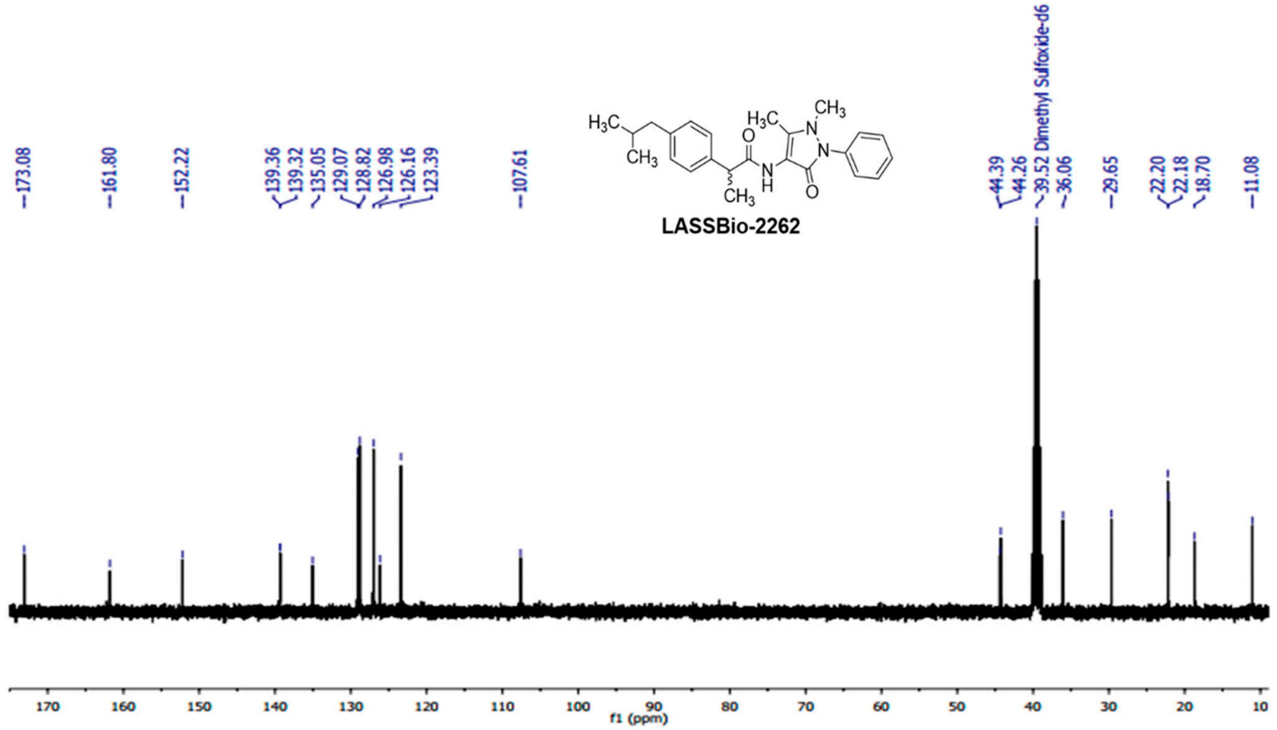

**Figure S2.** NMR spectrum¹³C pyrazolamide **(7)** (50 MHz, DMSO-d6).

<Chromatogram>

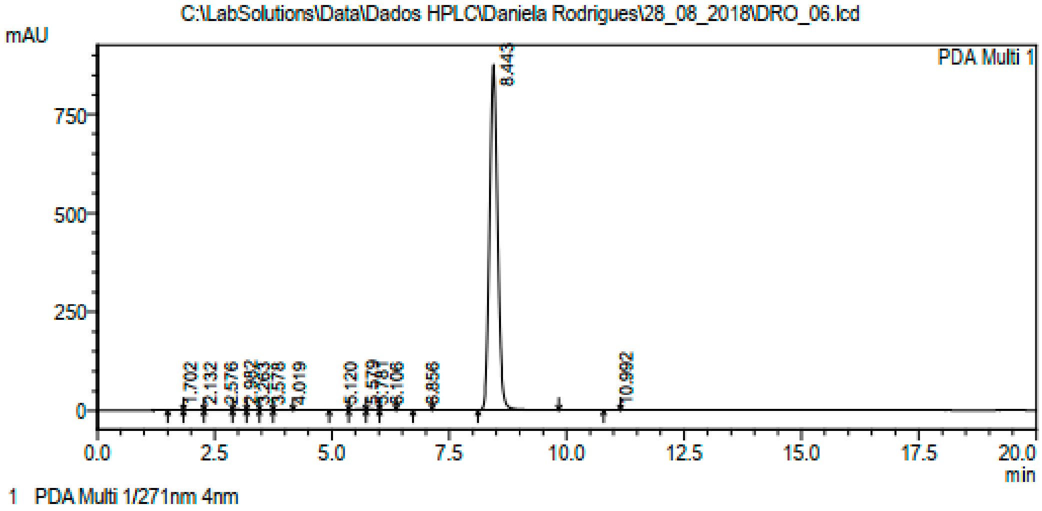


PeakTable

PDA Ch1 271nm 4nm

| Peak# | Ret. Time | Area | Height | Area % | Height % |
|---|---|---|---|---|---|
| 1 | 1.702 | 2255 | 242 | 0.022 | 0.027 |
| 2 | 2.132 | 5585 | 381 | 0.053 | 0.043 |
| 3 | 2.576 | 23552 | 995 | 0.225 | 0.112 |
| 4 | 2.982 | 14976 | 1971 | 0.143 | 0.222 |
| 5 | 3.263 | 5984 | 618 | 0.057 | 0.070 |
| 6 | 3.578 | 6686 | 763 | 0.064 | 0.086 |
| 7 | 4.019 | 6444 | 594 | 0.062 | 0.067 |
| 8 | 5.120 | 6749 | 742 | 0.065 | 0.084 |
| 9 | 5.579 | 30122 | 2219 | 0.288 | 0.250 |
| 10 | 5.781 | 20791 | 1824 | 0.199 | 0.206 |
| 11 | 6.106 | 7340 | 705 | 0.070 | 0.079 |
| 12 | 6.856 | 1699 | 169 | 0.016 | 0.019 |
| 13 | 8.443 | 10316978 | 875693 | 98.709 | 98.712 |
| 14 | 10.992 | 2705 | 203 | 0.026 | 0.023 |
| Total | | 10451867 | 887119 | 100.000 | 100.000 |

**Figure S3.** Chromatogram obtained for pyrazolamide **(7)**. Conditions: Shimadzu – LC20AD; Column: Kromasil 100-5 C18 250-4.6 mm; Mobile phase: 60% ACN, 40% water; Flow: 1mL/min; Detector: SPD-M20A (Diode Array); Wavelength: 271 nm.

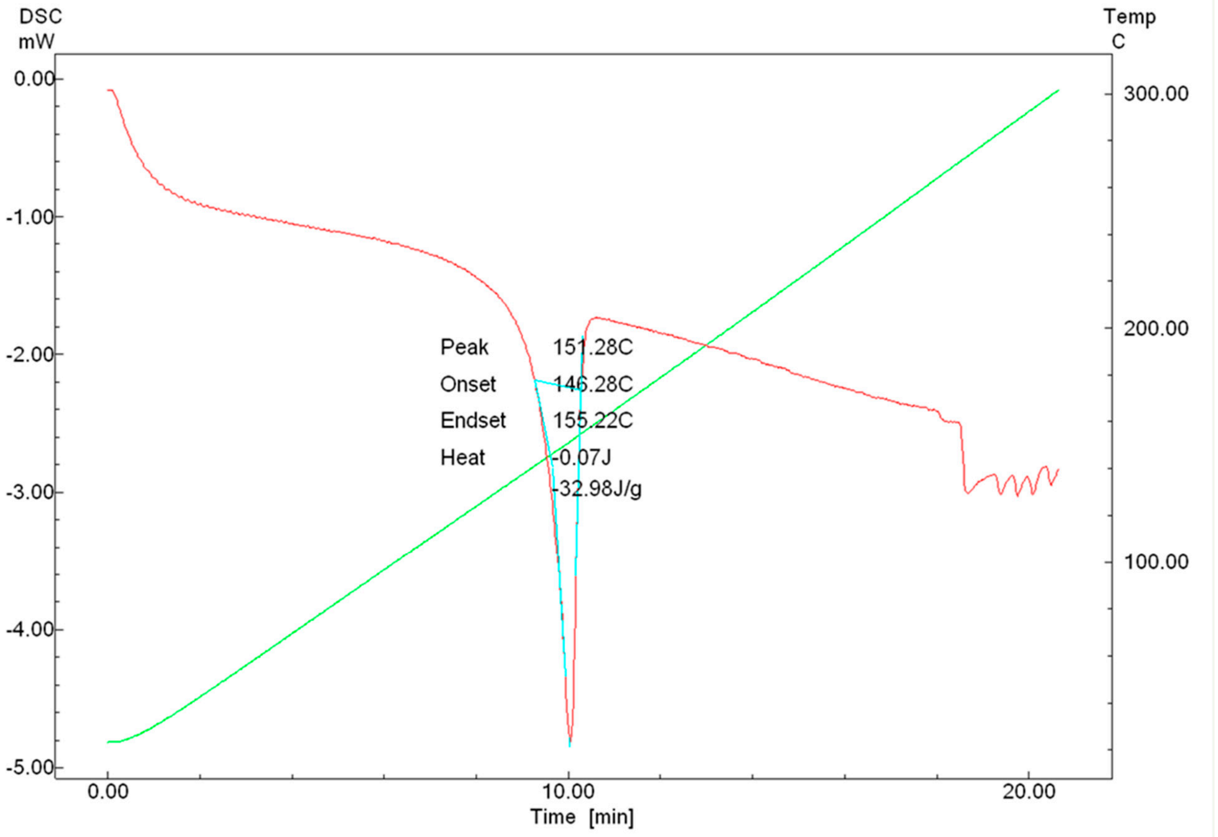


**Figure S4.** Determination of the melting point (FP) of pyrazolamide (**7**) determined by *Differential Scanning Calorimetry* (DSC) performed in a Shimadzu equipment (Model DSC-60).

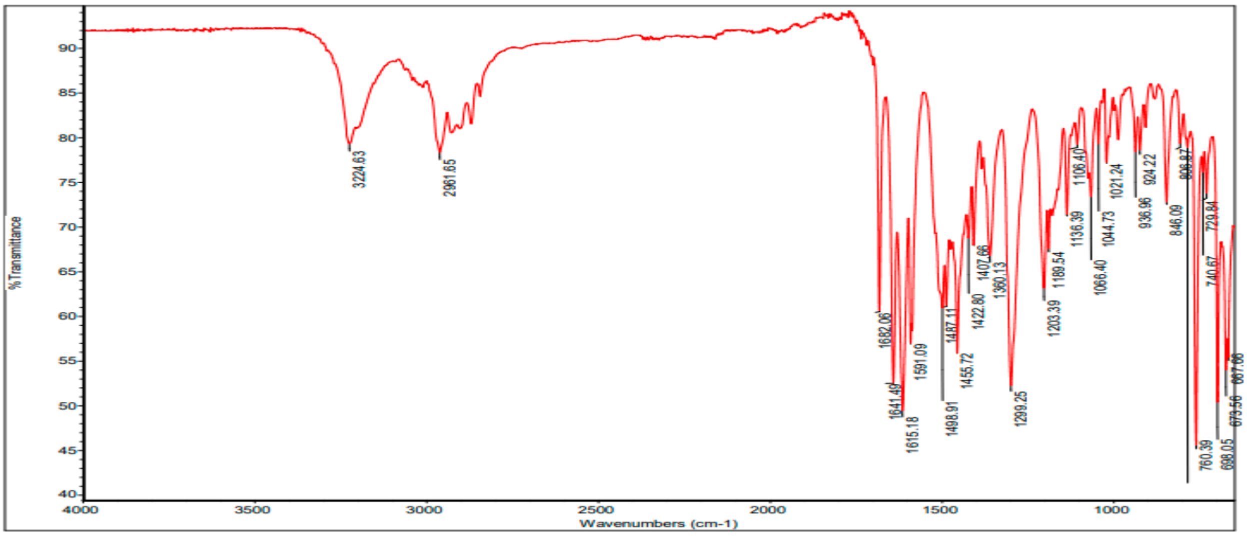


**Figure S5.** Infrared (ATR) of pyrazolamide (**7**).

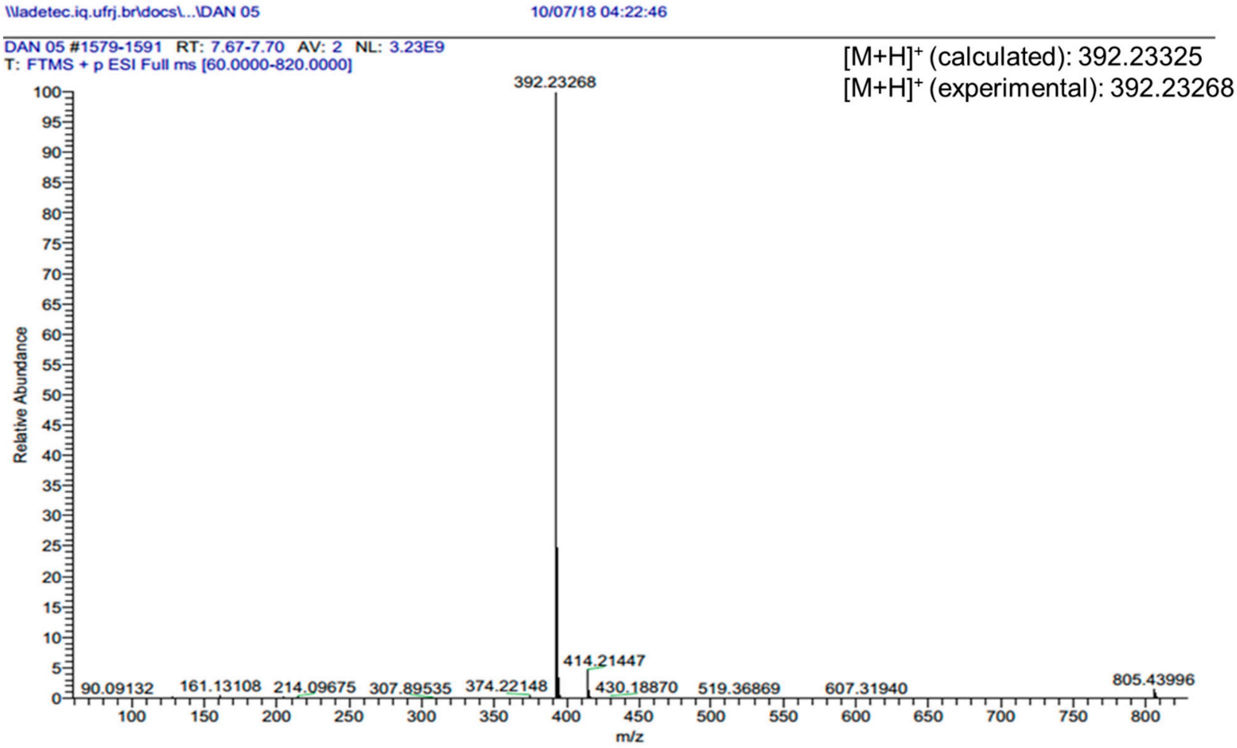


**Figure S6.** High resolution mass spectrum of pyrazolamide (**7**). Detection in positive mode ([M+H]+).

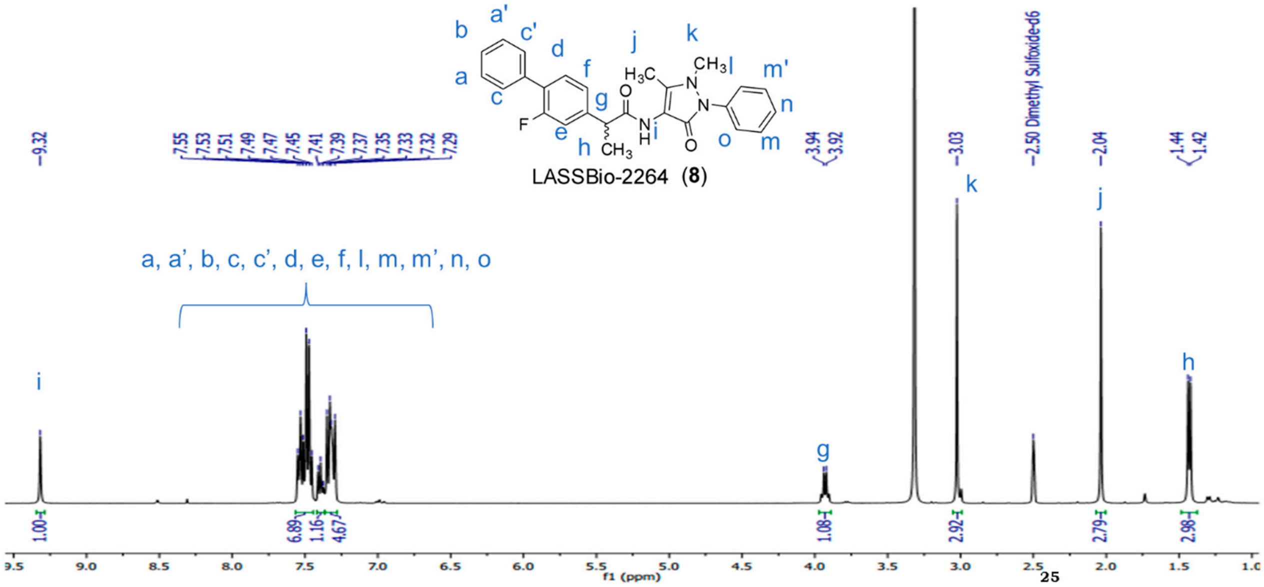


**Figure S7.** 1H NMR spectrum of pyrazolamide (**8**) (400 MHz, DMSO-d6).

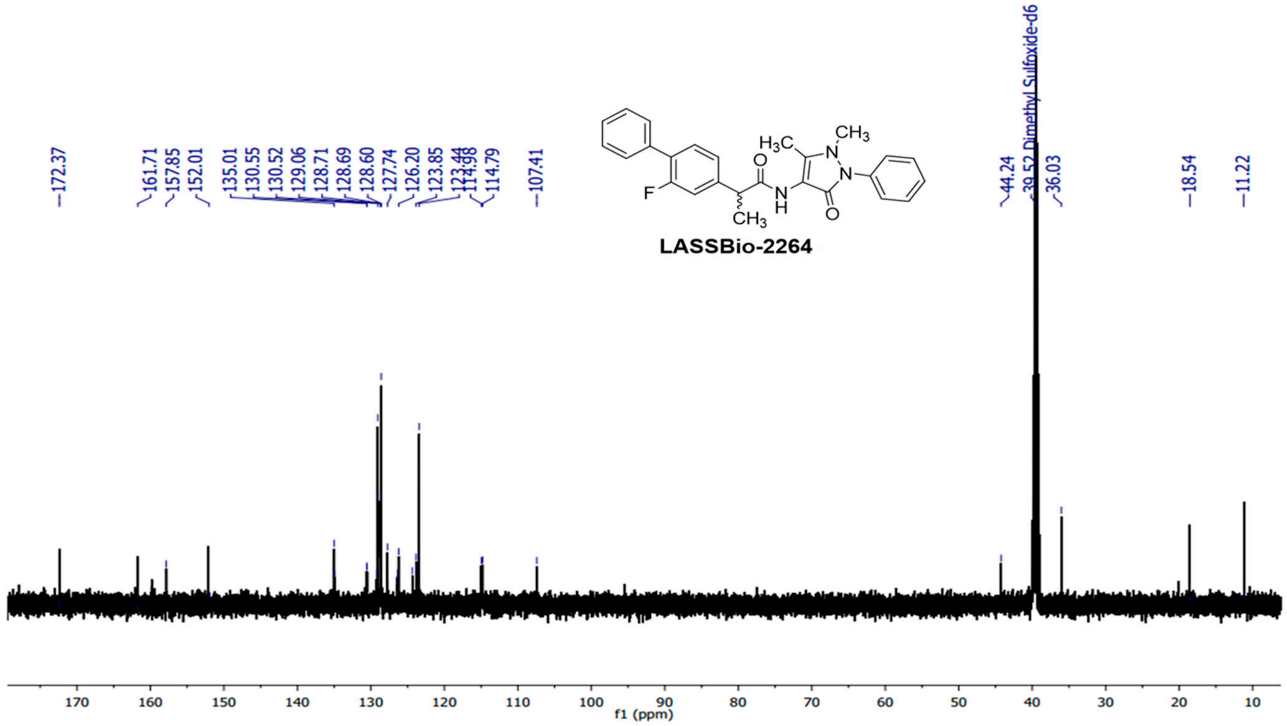


**Figure S8.** NMR spectrum $^{13}C$ pyrazolamide (**8**) (50 MHz, DMSO-d6).

**<Chromatogram>**

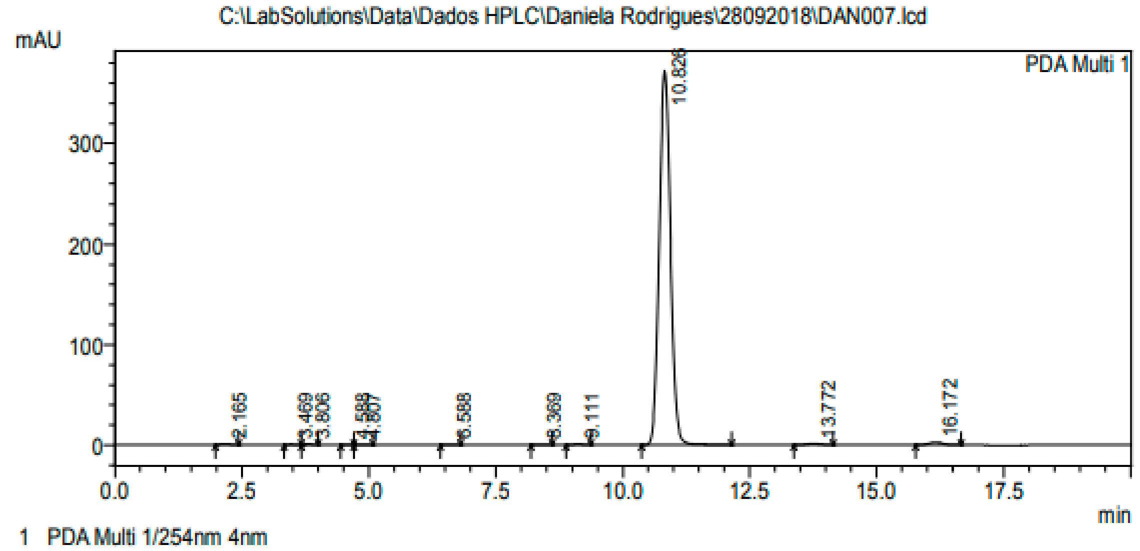


PeakTable

PDA Ch1 254nm 4nm

| Peak# | Ret. Time | Area | Height | Area % | Height % |
|---|---|---|---|---|---|
| 1 | 2.165 | 10192 | 740 | 0.174 | 0.195 |
| 2 | 3.469 | 3409 | 474 | 0.058 | 0.125 |
| 3 | 3.806 | 6968 | 964 | 0.119 | 0.254 |
| 4 | 4.588 | 1115 | 148 | 0.019 | 0.039 |
| 5 | 4.807 | 2137 | 222 | 0.036 | 0.059 |
| 6 | 6.588 | 3410 | 321 | 0.058 | 0.085 |
| 7 | 8.369 | 2188 | 179 | 0.037 | 0.047 |
| 8 | 9.111 | 9907 | 766 | 0.169 | 0.202 |
| 9 | 10.826 | 5757577 | 371270 | 98.024 | 97.920 |
| 10 | 13.772 | 24467 | 1332 | 0.417 | 0.351 |
| 11 | 16.172 | 52283 | 2740 | 0.890 | 0.723 |
| Total | | 5873653 | 379155 | 100.000 | 100.000 |

**Figure S9.** Chromatogram obtained for **pyrazolamide** (8). Conditions: Shimadzu – LC20AD; Column: Kromasil 100-5 C18 250-4.6 mm; Mobile phase: 60% ACN, 40% water; Flow: 1mL/min; Detector: SPD-M20A (Diode Array); Wavelength: 254 nm.

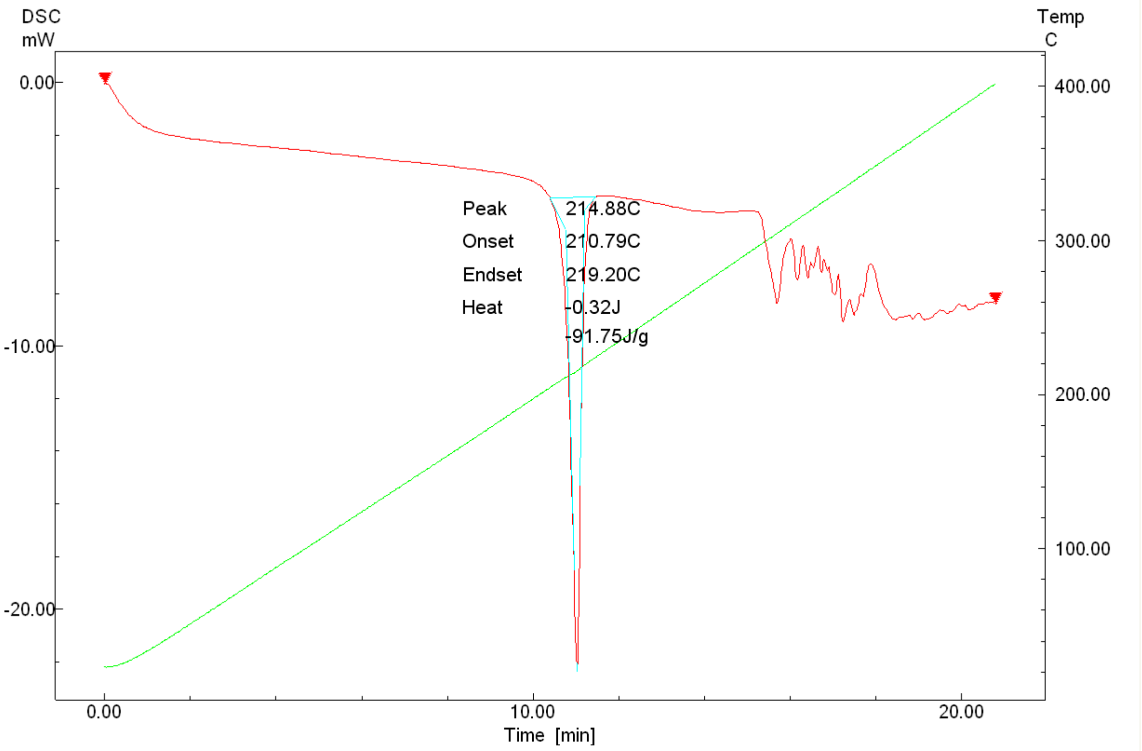


**Figure S10.** Determination of the melting point (FP) of pyrazolamide **(8)** determined by *Differential Scanning Calorimetry* (DSC) performed in a Shimadzu equipment (Model DSC-60).

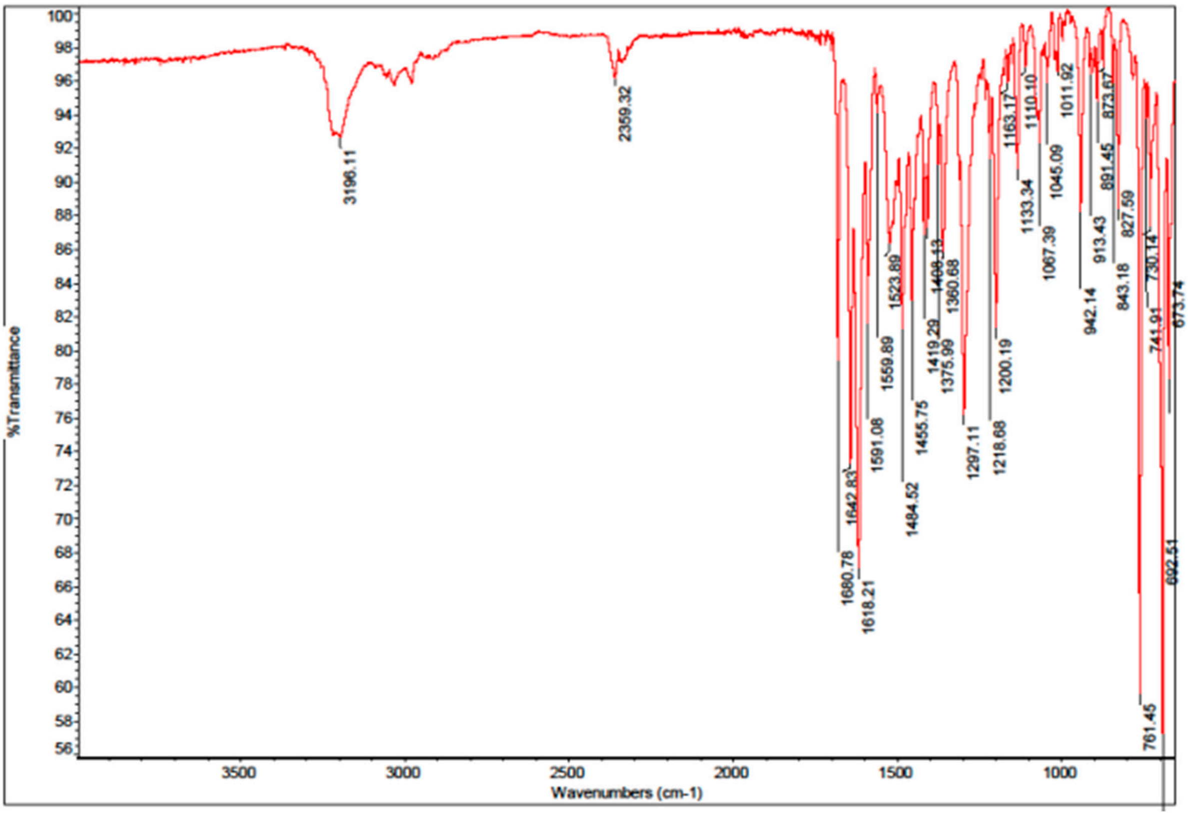


**Figure S11.** Infrared (ATR) of pyrazolamide (**8**).

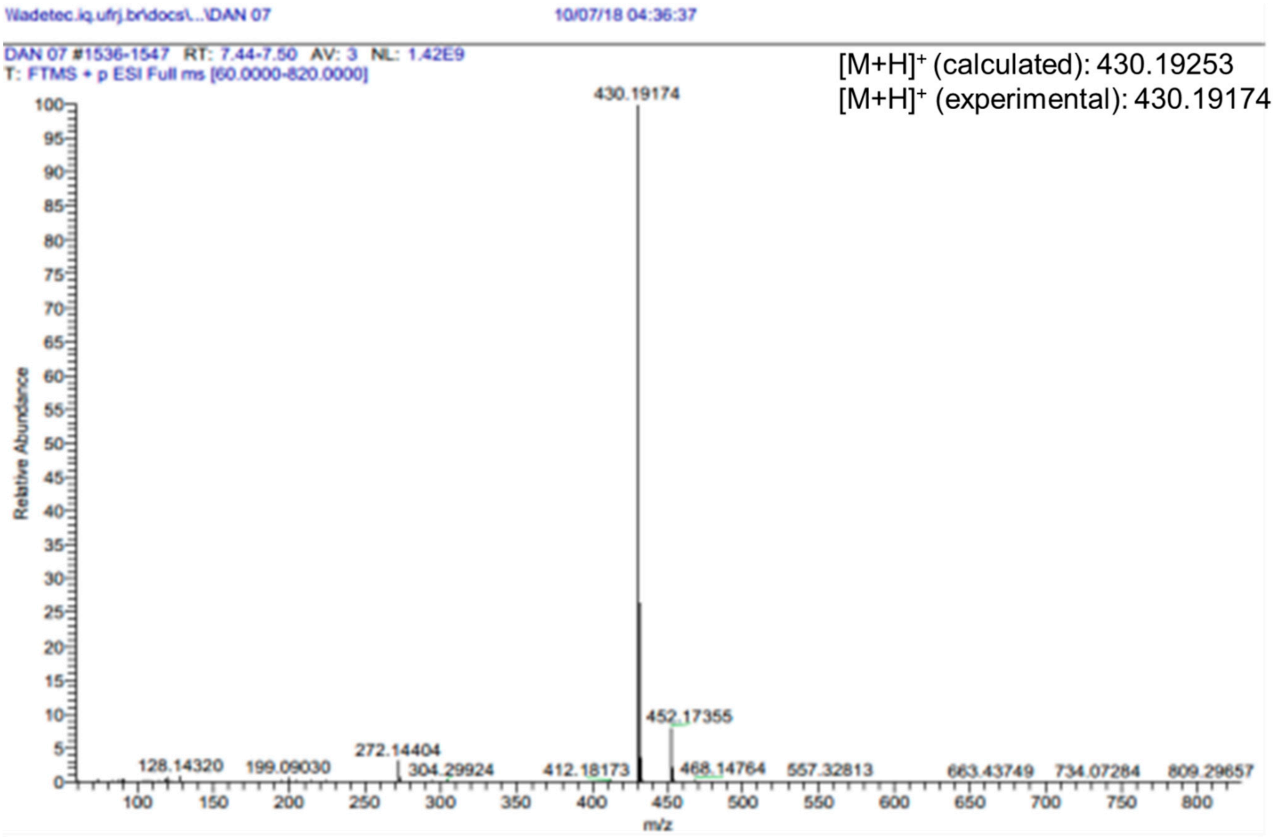


**Figure S12.** High resolution mass spectrum of pyrazolamide (**8**). Detection in positive mode ([M+H]+).

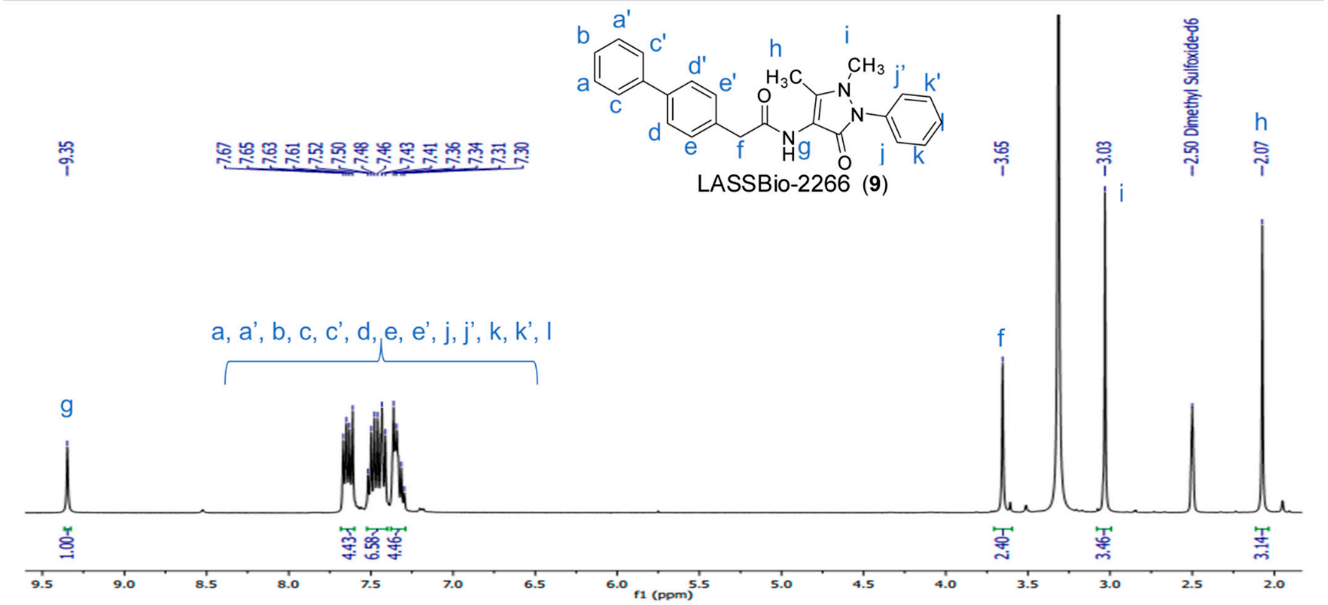


**Figure S13.** Spectrum $^1$H NMR pyrazolamide (**9**) (400 MHz, DMSO-d6).

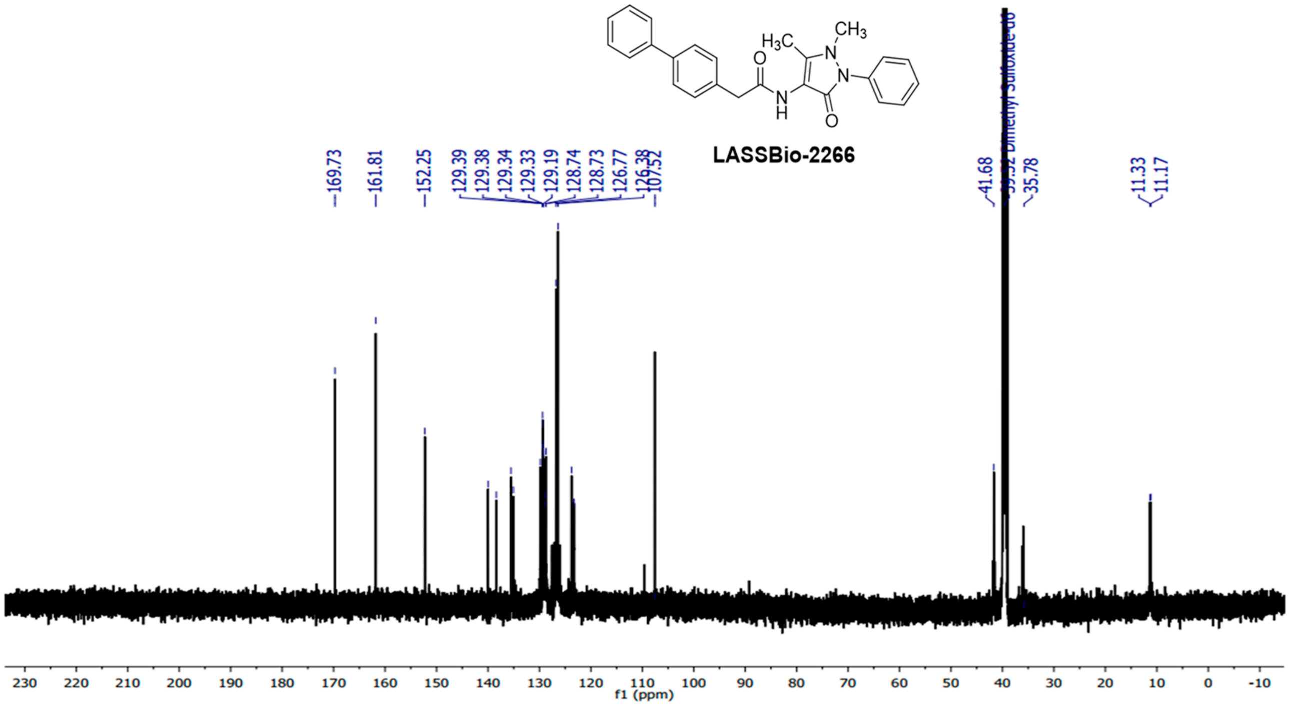


**Figure S14.** Spectrum $^{13}C$ NMR pyrazolamide **(9)** (50 MHz, DMSO-d6).

<Chromatogram>

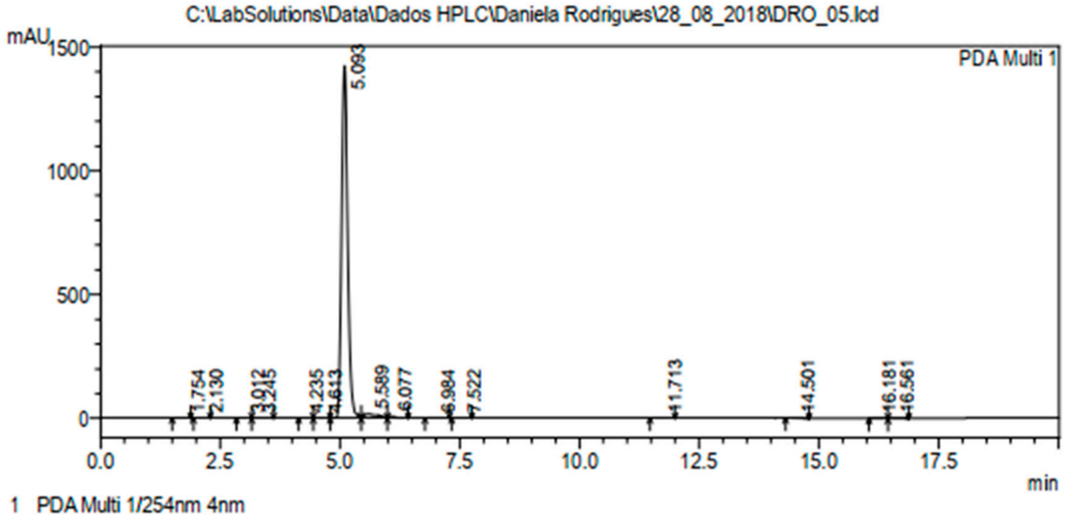


PeakTable

PDA Ch1 254nm 4nm

| Peak# | Ret. Time | Area | Height | Area % | Height % |
|---|---|---|---|---|---|
| 1 | 1.754 | 2871 | 492 | 0.022 | 0.034 |
| 2 | 2.130 | 3414 | 299 | 0.027 | 0.021 |
| 3 | 3.012 | 4384 | 372 | 0.034 | 0.026 |
| 4 | 3.245 | 6721 | 765 | 0.052 | 0.053 |
| 5 | 4.235 | 1421 | 111 | 0.011 | 0.008 |
| 6 | 4.613 | 2185 | 212 | 0.017 | 0.015 |
| 7 | 5.093 | 12443500 | 1423276 | 96.605 | 98.228 |
| 8 | 5.589 | 335900 | 15853 | 2.608 | 1.094 |
| 9 | 6.077 | 50987 | 5034 | 0.396 | 0.347 |
| 10 | 6.984 | 14408 | 1414 | 0.112 | 0.098 |
| 11 | 7.522 | 3283 | 312 | 0.025 | 0.022 |
| 12 | 11.713 | 4971 | 373 | 0.039 | 0.026 |
| 13 | 14.501 | 2567 | 176 | 0.020 | 0.012 |
| 14 | 16.181 | 1723 | 95 | 0.013 | 0.007 |
| 15 | 16.561 | 2521 | 166 | 0.020 | 0.011 |
| Total | | 12880854 | 1448949 | 100.000 | 100.000 |

**Figure S15.** Chromatogram obtained for pyrazolamide (**9**). Conditions: Shimadzu – LC20AD; Column: Kromasil 100-5 C18 250-4.6 mm; Mobile phase: 60% ACN, 40% water; Flow: 1mL/min; Detector: SPD-M20A (Diode Array); Wavelength: 254 nm.

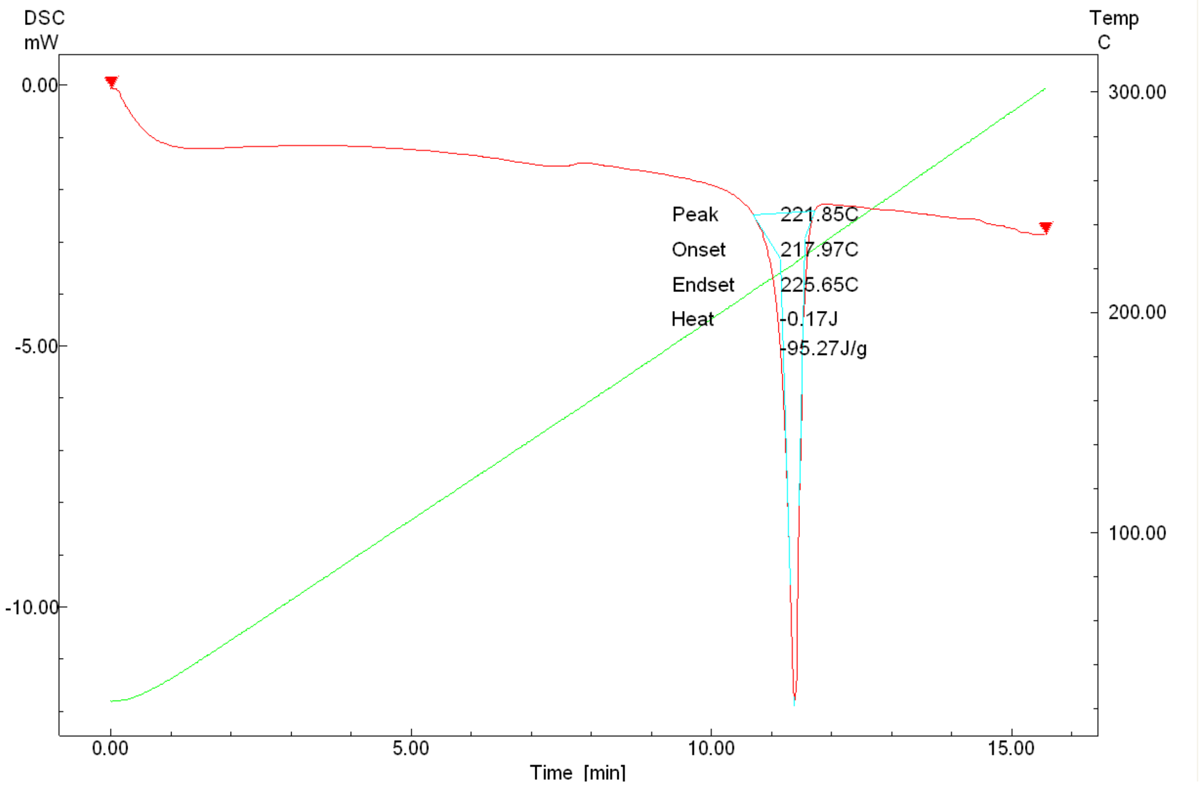


**Figure S16.** Determination of the melting point (FP) of pyrazolamide **(9)** determined by *Differential Scanning Calorimetry* (DSC) performed in a Shimadzu equipment (Model DSC-60).

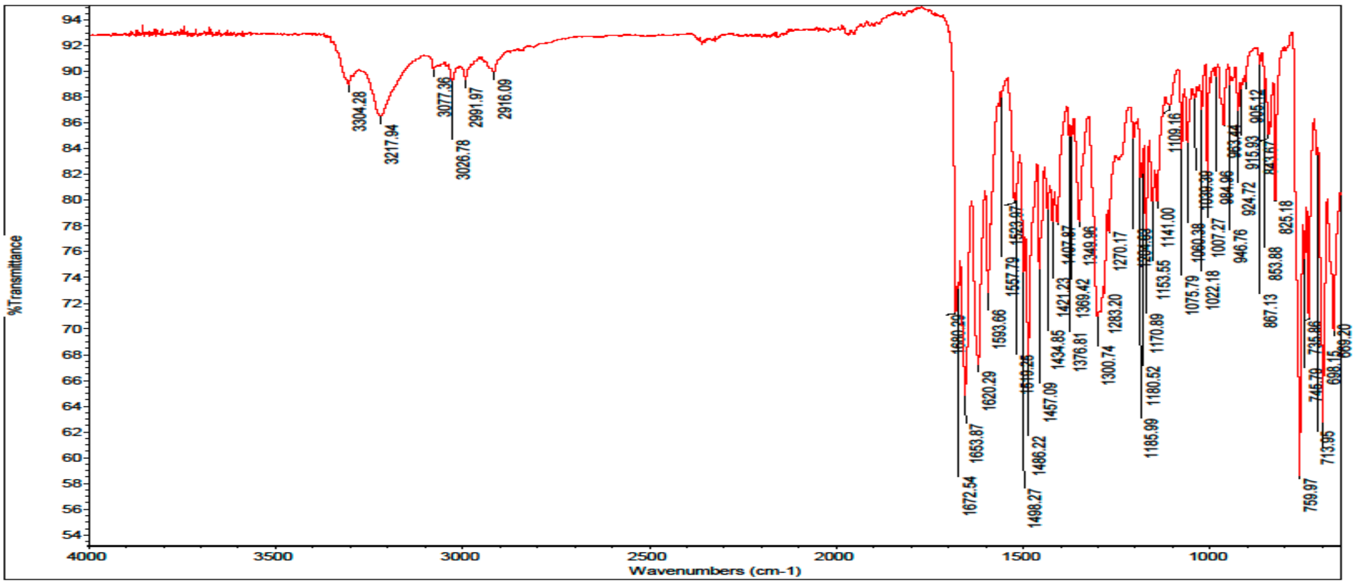


**Figure S17.** Infrared (ATR) pyrazolamide **(9)**.

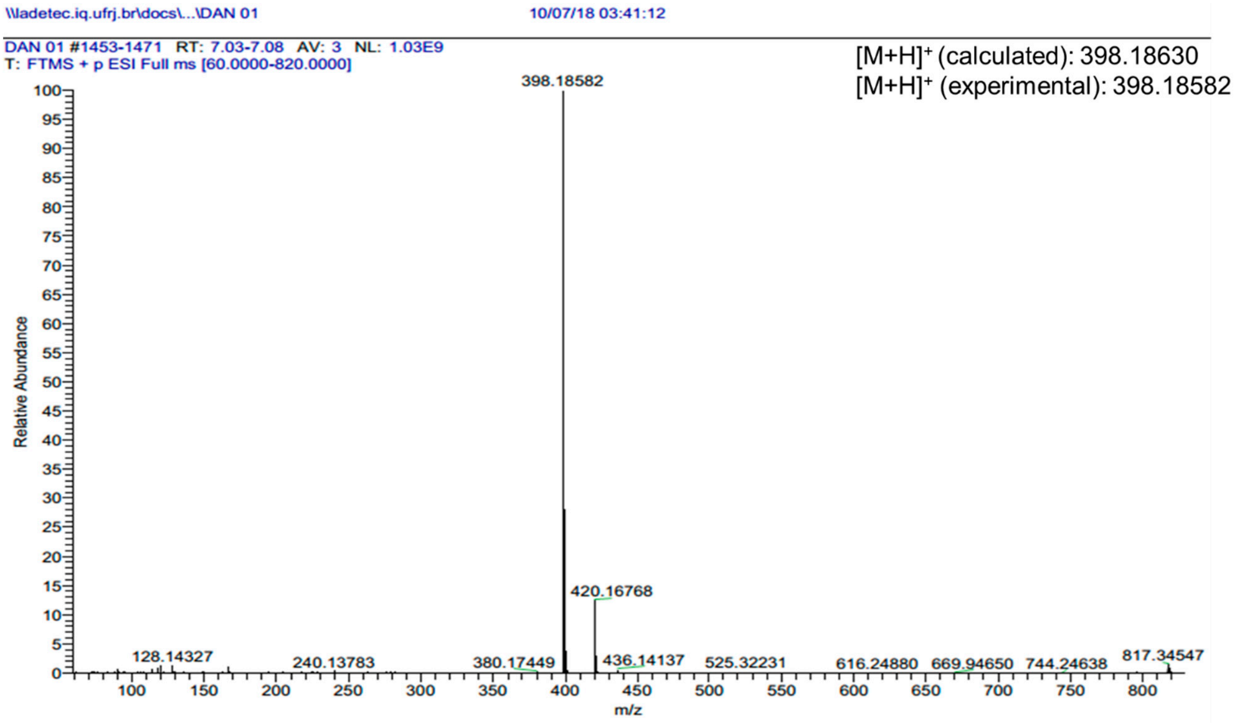


**Figure S18.** High resolution mass spectrum of pyrazolamide **(9)**. Detection in positive mode ($[M+H]^+$).

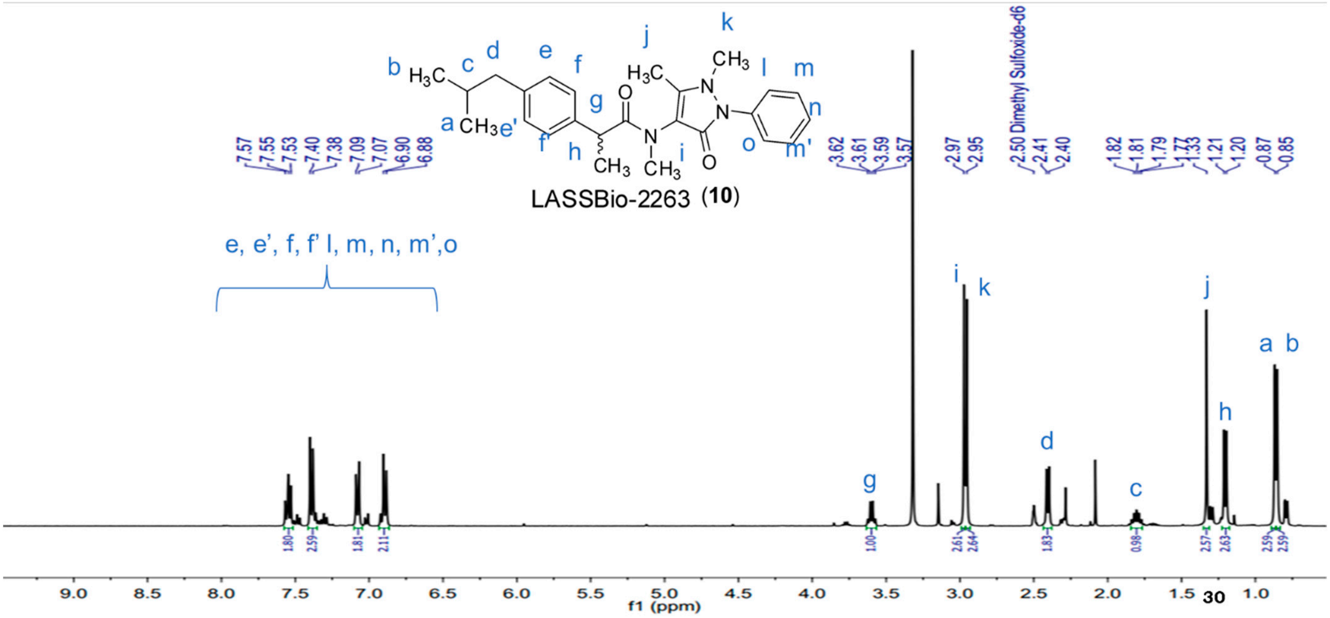


**Figure S19.** $^{1}H$ NMR spectrum of pyrazolamide (**10**) (400 MHz, DMSO-d6).

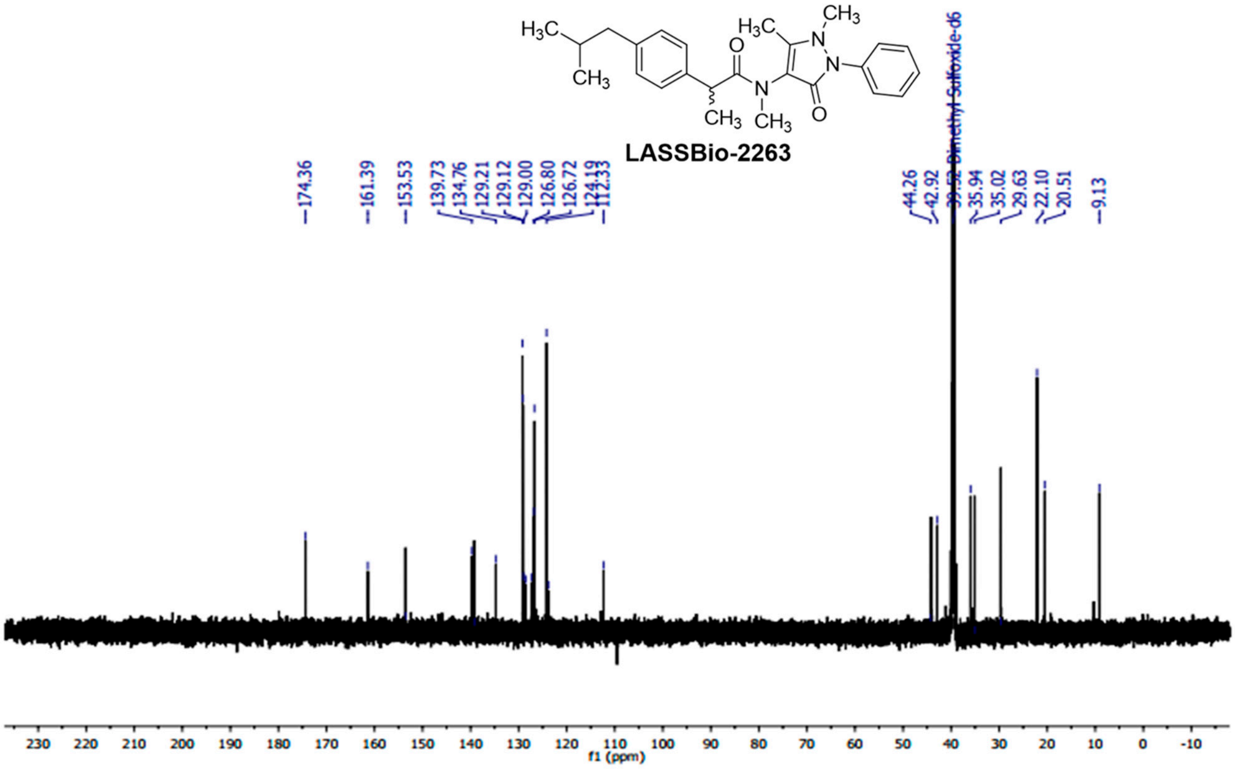


**Figure S20.** NMR spectrum $^{13}C$ of pyrazolamide (**10**) (50 MHz, DMSO-d6).

<Chromatogram>

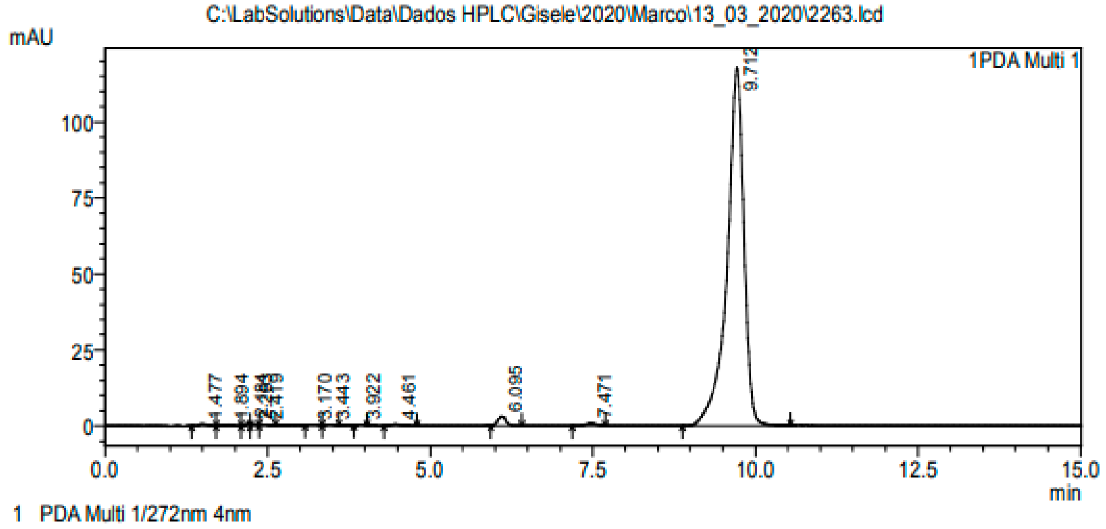


PeakTable

PDA Ch1 272nm 4nm

| Peak# | Ret. Time | Area | Height | Area % | Height % |
|---|---|---|---|---|---|
| 1 | 1.477 | 5457 | 600 | 0.248 | 0.479 |
| 2 | 1.894 | 2871 | 293 | 0.130 | 0.234 |
| 3 | 2.184 | 2958 | 901 | 0.134 | 0.719 |
| 4 | 2.263 | 3140 | 583 | 0.143 | 0.465 |
| 5 | 2.419 | 1917 | 221 | 0.087 | 0.177 |
| 6 | 3.170 | 2023 | 202 | 0.092 | 0.161 |
| 7 | 3.443 | 1637 | 239 | 0.074 | 0.191 |
| 8 | 3.922 | 2200 | 350 | 0.100 | 0.279 |
| 9 | 4.461 | 4727 | 416 | 0.215 | 0.332 |
| 10 | 6.095 | 26490 | 3082 | 1.204 | 2.460 |
| 11 | 7.471 | 9913 | 815 | 0.451 | 0.650 |
| 12 | 9.712 | 2136763 | 117604 | 97.121 | 93.854 |
| Total | | 2200096 | 125305 | 100.000 | 100.000 |

**Figure S21.** Chromatogram obtained for pyrazolamide (**10**). Conditions: Shimadzu – LC20AD; Column: Kromasil 100-5 C18 250-4.6 mm; Mobile phase: 60% ACN, 40% water; Flow: 1mL/min; Detector: SPD-M20A (Diode Array); Wavelength: 272 nm.

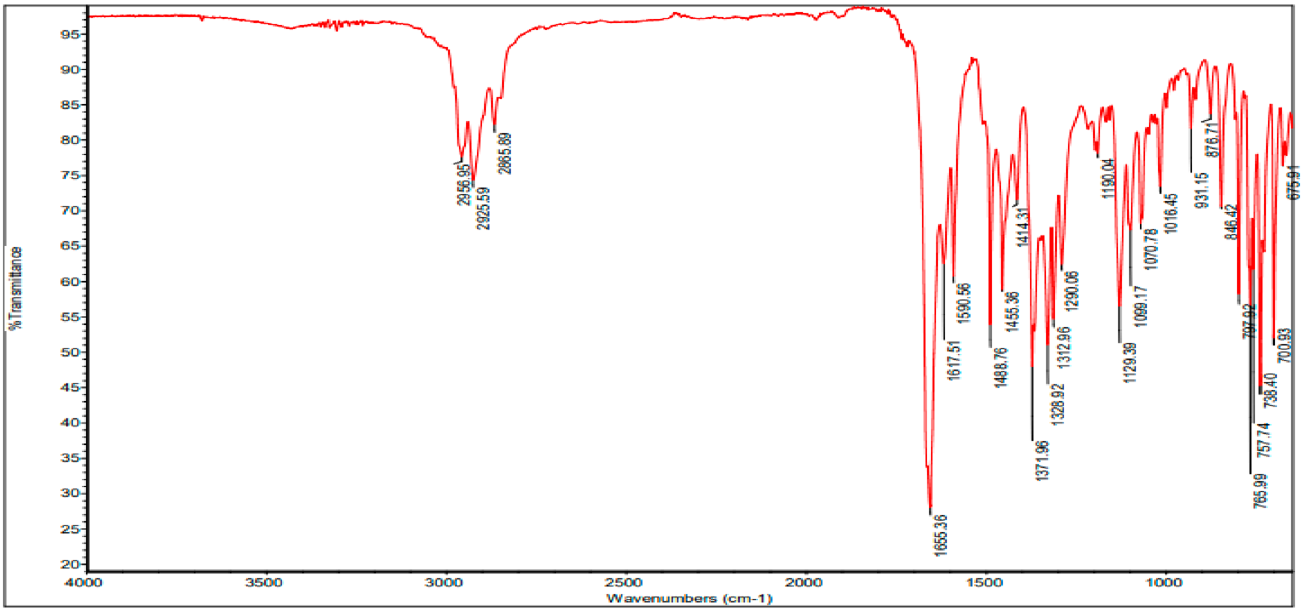


**Figure S22.** Infrared (ATR) pyrazolamide **(10)**.

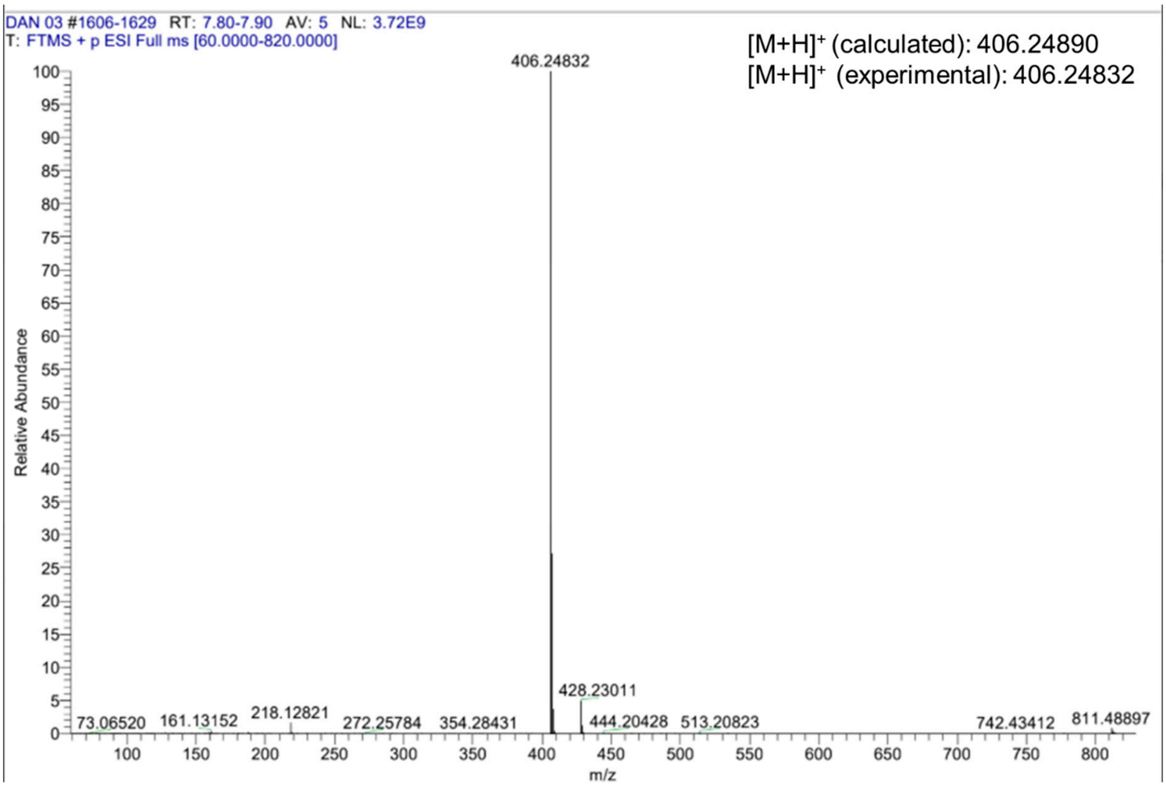


**Figure S23.** High resolution mass spectrum of pyrazolamide **(10)**. Detection in positive mode ($[M+H]^+$).

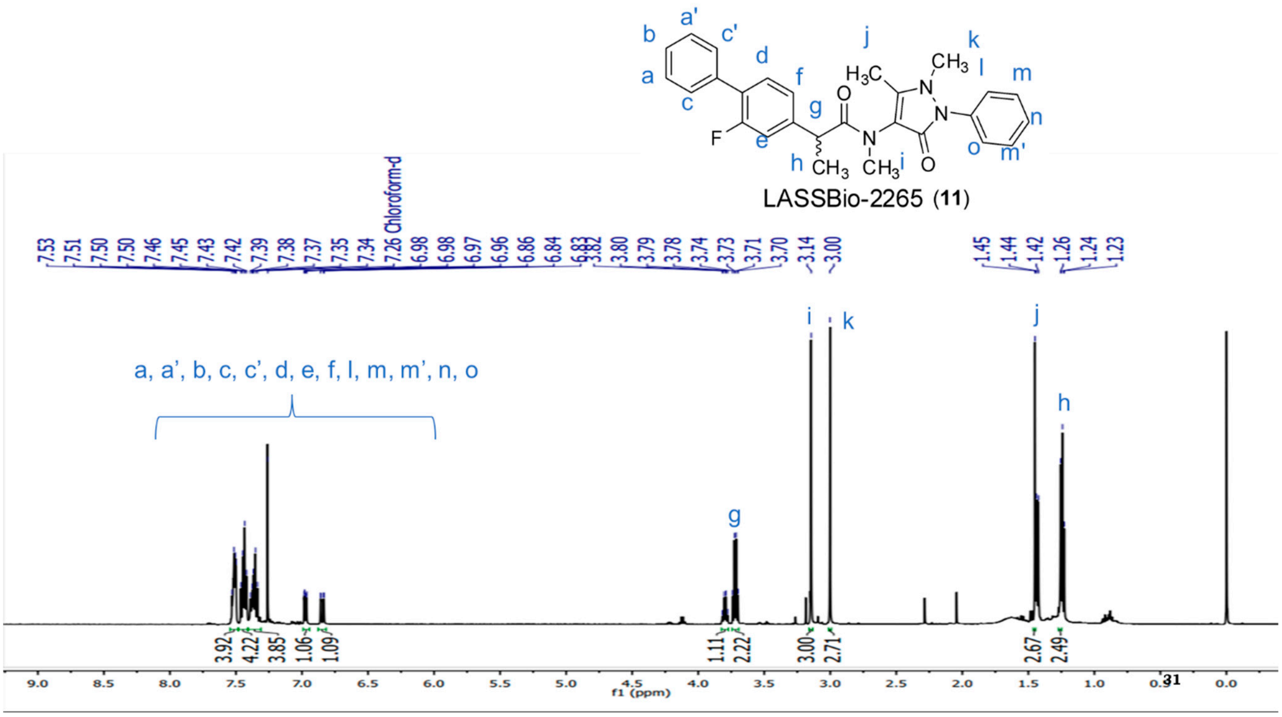

**Figure S24.** Spectrum of RMN$^{1}$H to **LASSBio-2265 (11)** (500 MHz, acetonitrile-*d*3).

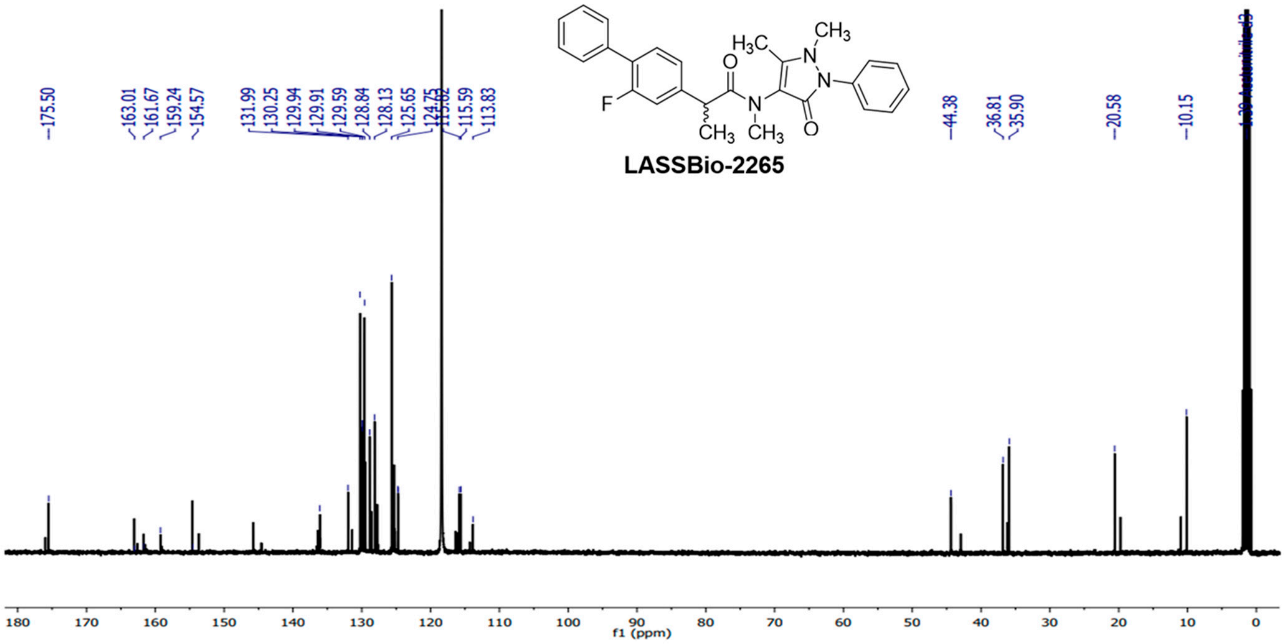

**Figure S25.** NMR spectrum $^{13}$C of **LASSBio-2265** (**11**) (100 MHz, $CDCl_3$).

<Chromatogram>

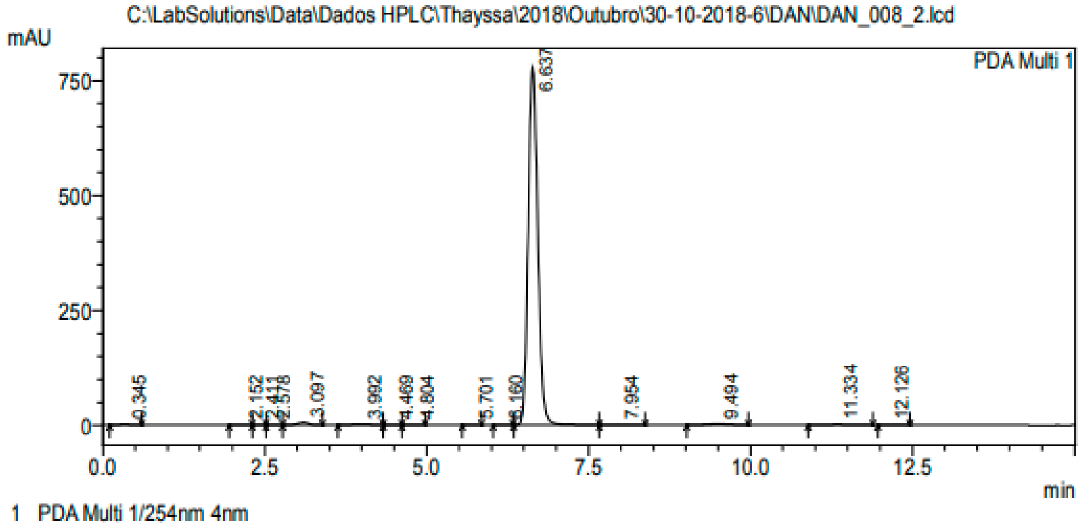


PeakTable

PDA Ch1 254nm 4nm

| Peak# | Ret. Time | Area | Height | Area % | Height % |
|---|---|---|---|---|---|
| 1 | 0.345 | 16092 | 1310 | 0.194 | 0.165 |
| 2 | 2.152 | 8175 | 613 | 0.098 | 0.077 |
| 3 | 2.411 | 2178 | 220 | 0.026 | 0.028 |
| 4 | 2.578 | 2817 | 355 | 0.034 | 0.045 |
| 5 | 3.097 | 70327 | 5795 | 0.846 | 0.732 |
| 6 | 3.992 | 32786 | 1689 | 0.394 | 0.213 |
| 7 | 4.469 | 1933 | 131 | 0.023 | 0.017 |
| 8 | 4.804 | 2867 | 344 | 0.034 | 0.043 |
| 9 | 5.701 | 1696 | 201 | 0.020 | 0.025 |
| 10 | 6.160 | 1027 | 105 | 0.012 | 0.013 |
| 11 | 6.637 | 8083227 | 776741 | 97.248 | 98.123 |
| 12 | 7.954 | 11066 | 542 | 0.133 | 0.069 |
| 13 | 9.494 | 55177 | 2446 | 0.664 | 0.309 |
| 14 | 11.334 | 19397 | 888 | 0.233 | 0.112 |
| 15 | 12.126 | 3201 | 224 | 0.039 | 0.028 |
| Total | | 8311964 | 791603 | 100.000 | 100.000 |

**Figure S26.** Chromatogram obtained for **LASSBio-2265** (**11**). Conditions: Shimadzu – LC20AD; Column: Kromasil 100-5 C18 250-4.6 mm; Mobile phase: 60% ACN, 40% water; Flow: 1mL/min; Detector: SPD-M20A (Diode Array); Wavelength: 254 nm.

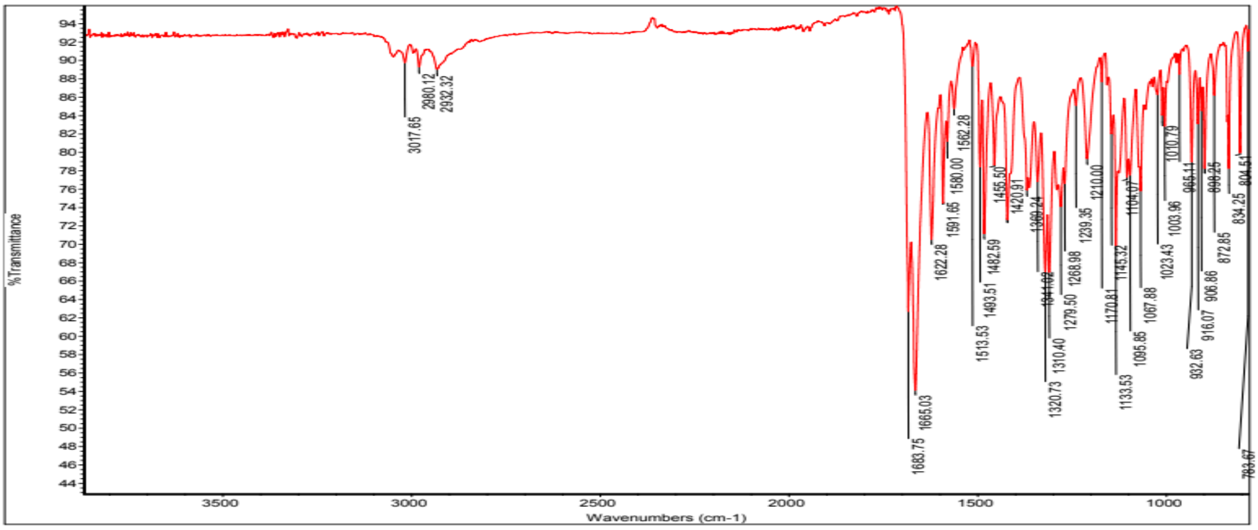


**Figure S27.** Infrared (ATR) of **LASBio-2265** (**11**).

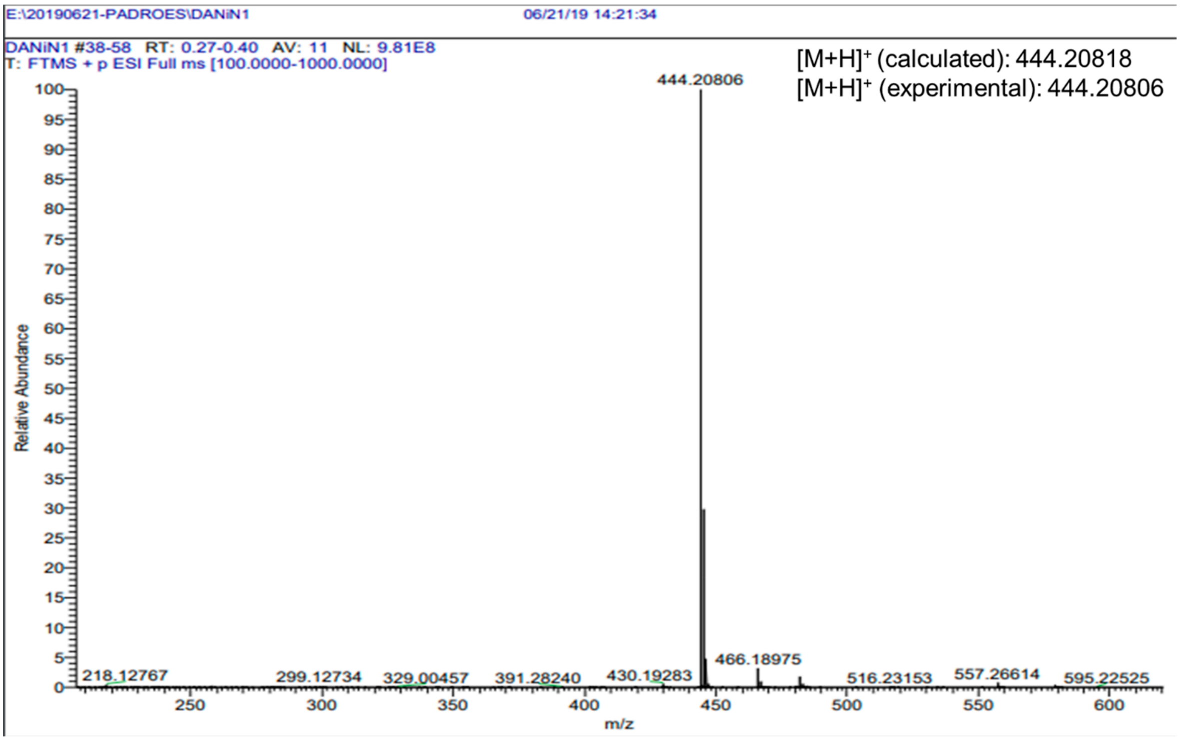


**Figure S28.** High resolution mass spectrum of **LASSBio-2265** (**11**). Detection in positive mode ([M+H]+).

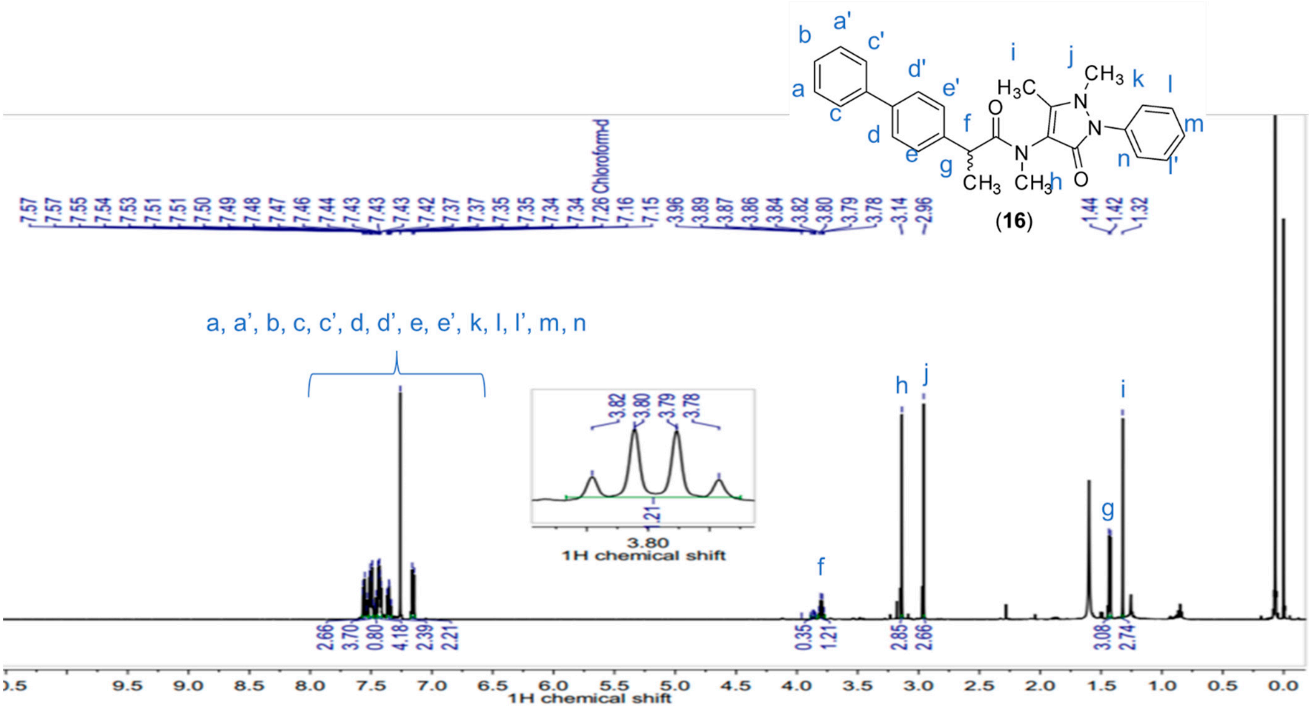


**Figure S29.** $^{1}$H NMR spectrum[1]of bismethylated pyrazolamide **(16)** (400 MHz, $CDCl_3$).

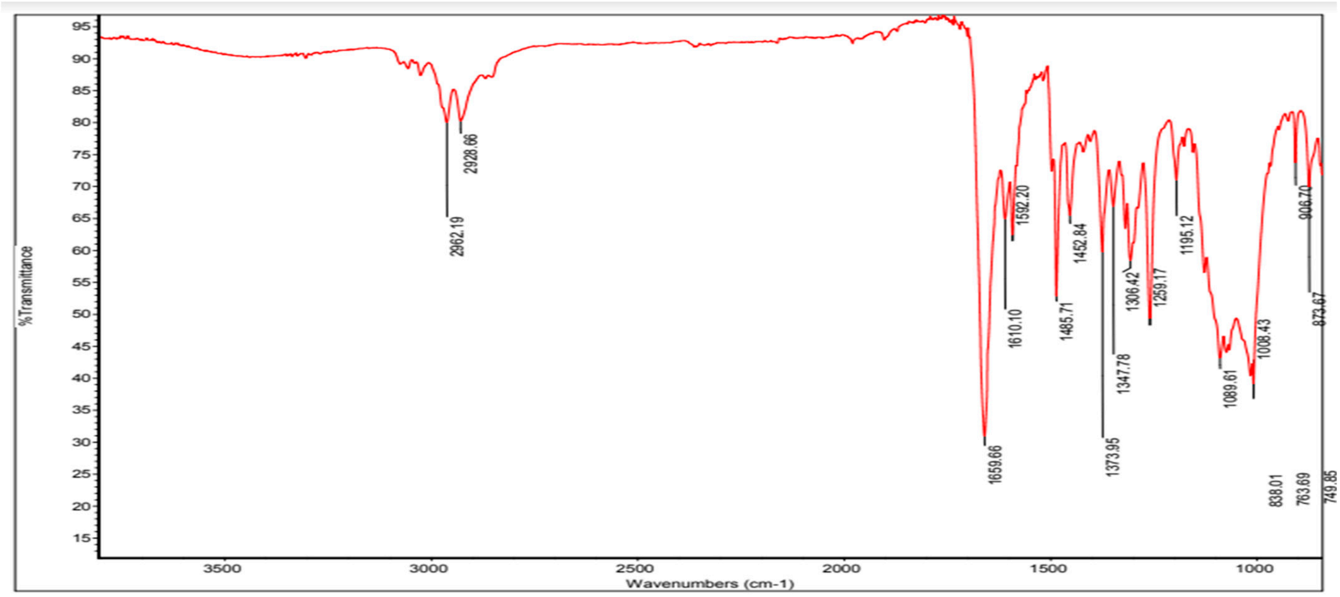


**Figure S30.** Infrared (ATR) of bismethylated pyrazolamide **(16)**.

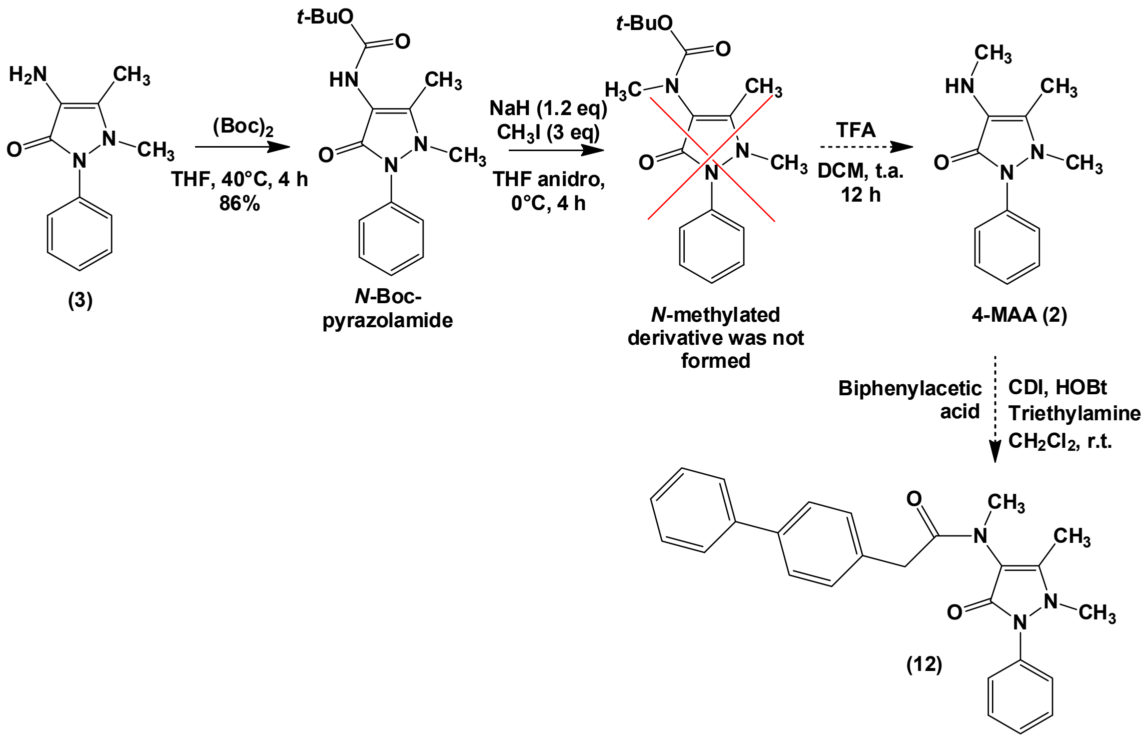


**Figure S31**. Alternative methodology tried for the synthesis of pyrazolamide (**12**).

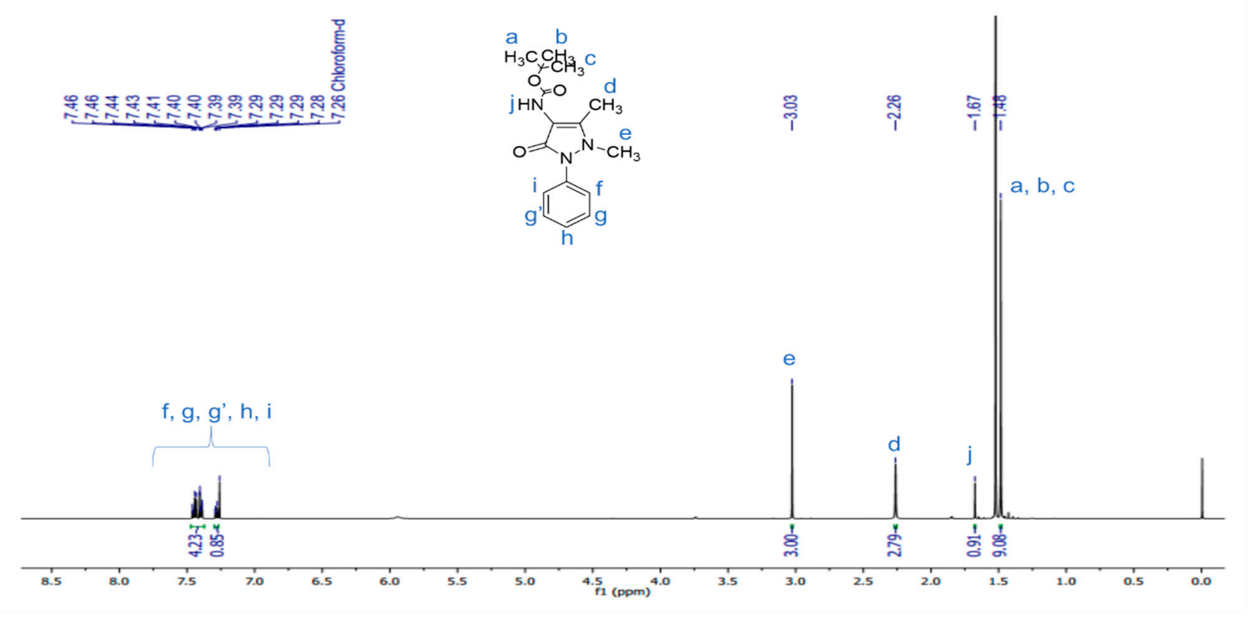


**Figura S32**. $^1$H NMR spectrum[1]of N-Boc-pyrazolamide (500 MHz, $CDCl_3$).

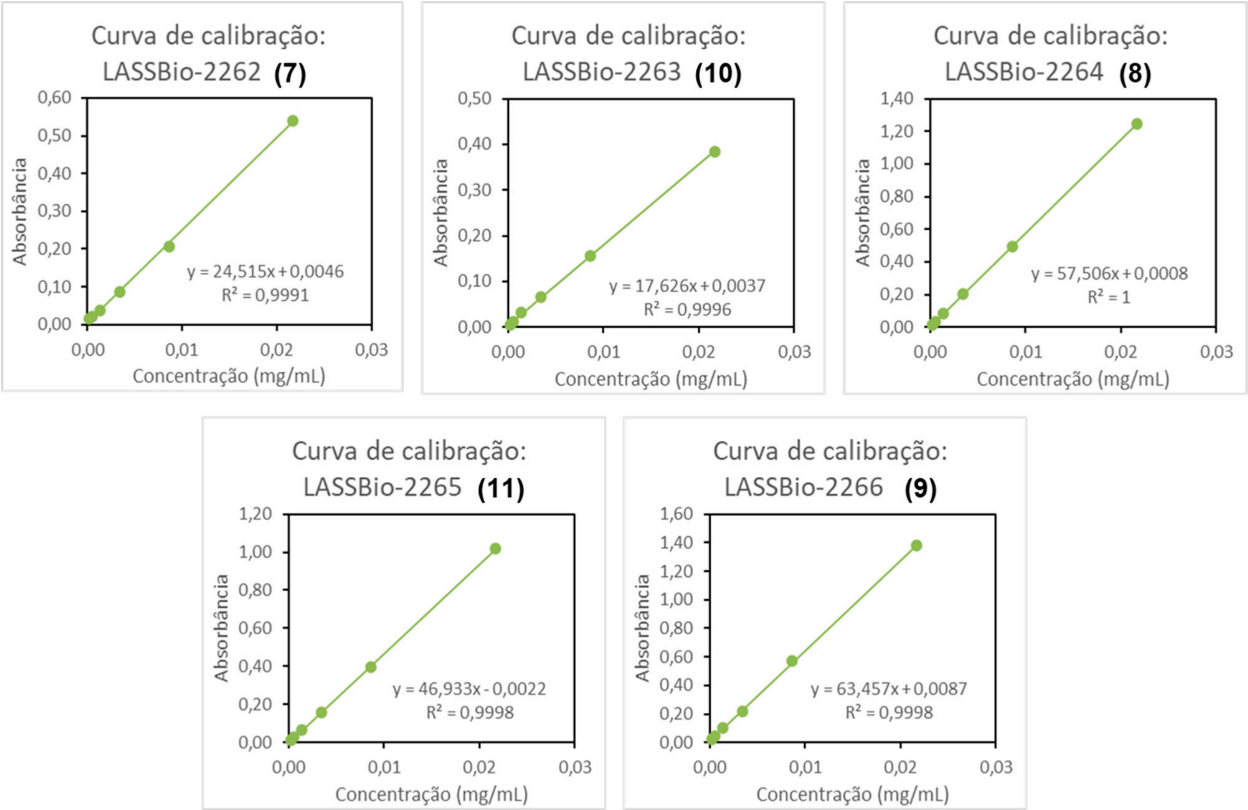


**Figure S33.** Calibration curves for determination of aqueous solubility of the pyrazolamides (**7-11**).

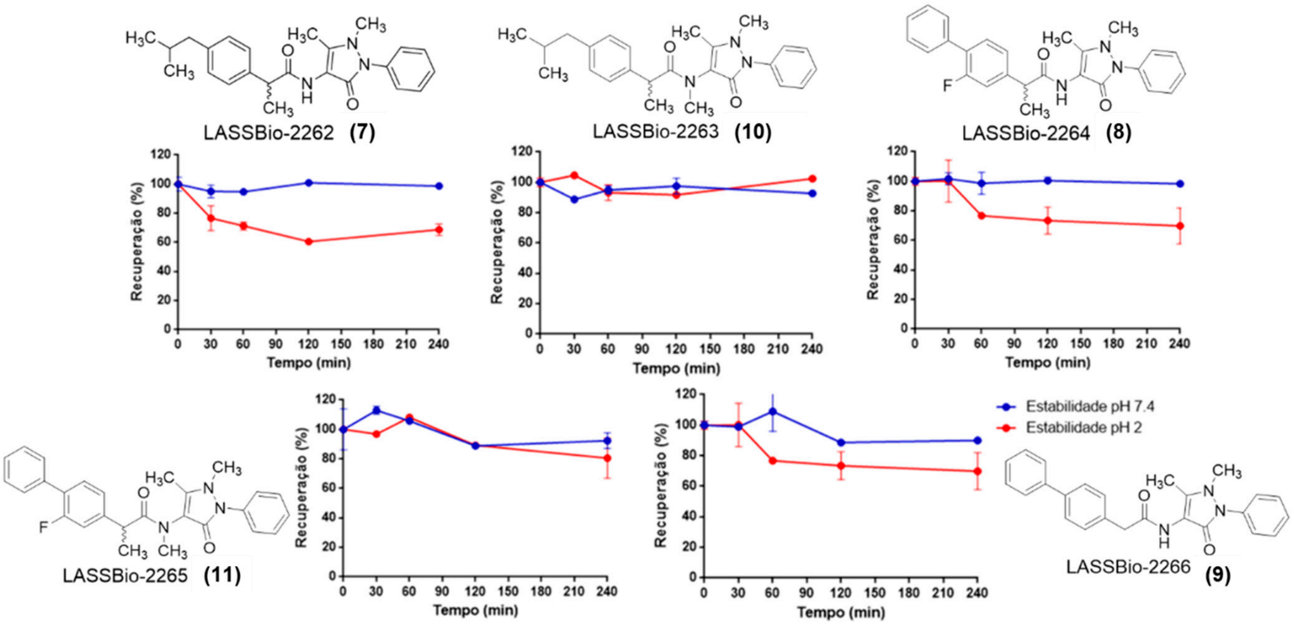


**Figure S34.** Chemical stability of pyrazolamides (**7-11**) at pH 2 (red) and pH 7.4 ( blue). Experiments performed in triplicate. Reading performed in HPLC-PDA (Shimadzu-LC20A; Kromasil C-18 column (4.6mm x 250mm); SPD-M20A detector (Diode Array); flow: 1ml/minute]; Mobile phase: Acetonitrile:water, 40%. Wavelength: 272 nm (**LASSBio-2262** (**7**) and **LASSBio-2263** (**10**)), 247 nm (**LASSBio-2264** (**8**) and **LASSBio-2265** (**11**)) and 253 nm (**LASSBio-2266** (**9**)) The calculations were performed according to the peak area values of the analytes.

**Table S1.** Absorbed fraction of drugs used as standard and pyrazolamides **(7-11)** determined in the PAMPA-GTI model.

| **COMPOUND** | **Pe** Lit ($10^{-6}$cm/s)** | **Pe** Exp ($10^{-6}$ cm/s)** | **Fa# (%)** | **Classification** |
|---|---|---|---|---|
| Acyclovir* | 0,06 | 0,08 | 2,98 | LOW |
| Atenolol* | 0,1 | 0,11 | 4,22 | LOW |
| Ranitidine* | 0,5 | 0,38 | 13,48 | LOW |
| Sulfassalazine* | 0,3 | 0,45 | 15,5 | LOW |
| Aspirin* | 3,8 | 1,69 | 47,36 | AVERAGE |
| Hydrocortisone* | 3,4 | 2,16 | 55,93 | AVERAGE |
| Prednisone* | 5,7 | 3,09 | 69,16 | AVERAGE |
| Ketoconazole* | 3,3 | 4,93 | 84,68 | HIGH |
| Coumarin* | 22,9 | 22,65 | 99,98 | HIGH |
| Diclofenac* | 12,5 | 13,01 | 99,28 | HIGH |
| Verapamil* | 7,4 | 6,45 | 91,40 | HIGH |
| **(7)** | **-** | **15,38** | **99,71** | **HIGH** |
| **(10)** | **-** | **9,15** | **96,91** | **HIGH** |
| **(8)** | **-** | **9,69** | **97,49** | **HIGH** |
| **(11)** | **-** | **10,18** | **97,91** | **HIGH** |
| **(9)** | **-** | **11,00** | **98,47** | **HIGH** |

*Drugs used as standard for experimental validation. **Pe= permeability coefficient; #Fa= absorbed fraction (ZHU et al., 2002).

**Table S2.** Permeability of drugs used as standard, and pyrazolamides **(7-11)** determined in the PAMPA-BBB model.

| **Compound** | **Pe literatura (10-6 cm s-1)** | **Pe experimental (10-6 cm s-1)** | **Classification** |
|---|---|---|---|
| **Atenolol** | **0,8** | **0,46** | **CNS -** |
| **Caffeine** | **1,3** | **0,81** | **CNS -** |
| **Diazepam** | **16** | **14,30** | **CNS +** |
| **Enoxacin** | **0,9** | **0,53** | **CNS -** |
| **Ofloxacin** | **0,8** | **1,29** | **CNS -** |
| **Testosterone** | **17** | **16,85** | **CNS +** |
| **Verapamil** | **16** | **15,40** | **CNS +** |
| **(7)** | **-** | **12,77** | **CNS +** |
| **(10)** | **-** | **12,68** | **CNS +** |
| **(8)** | **-** | **17,25** | **CNS +** |
| **(11)** | **-** | **9,26** | **CNS +** |
| **(9)** | **-** | **4,51** | **CNS +** |

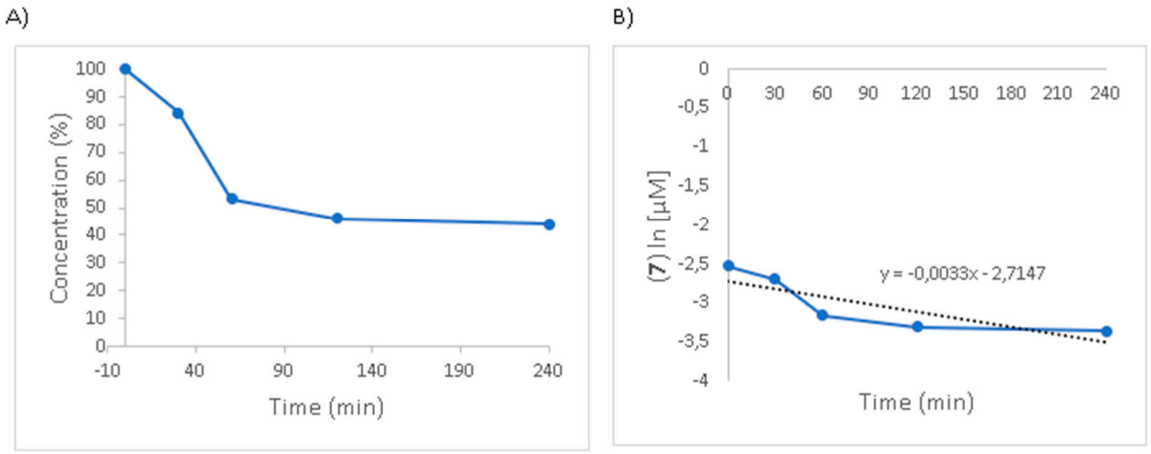


**Figure S35.** A) Plasma stability profile of (**7**); B) First order rate constant (k) for elimination.

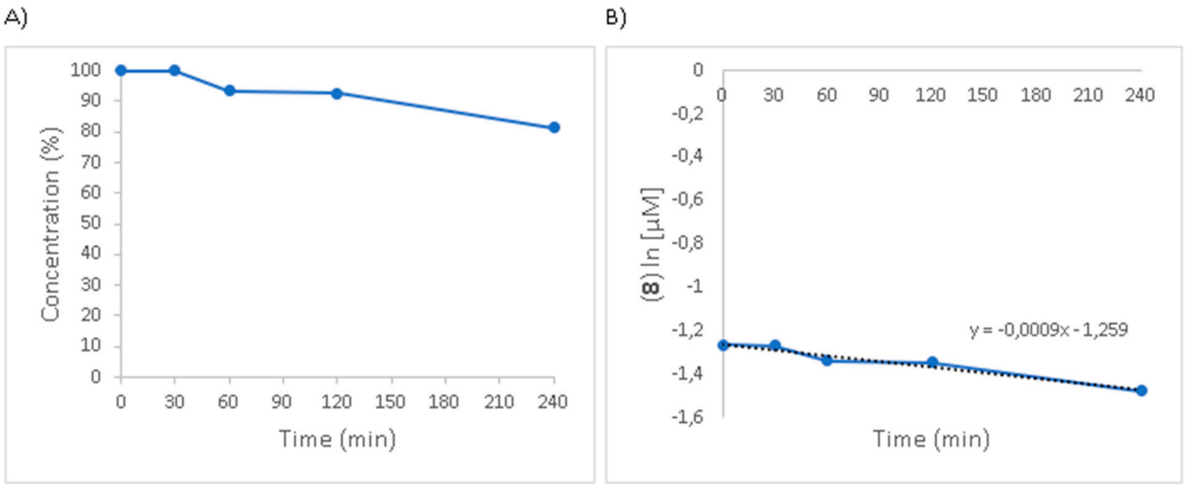


**Figure S36.** A) Plasma stability profile of (**8**); B) First order rate constant (k) for elimination.

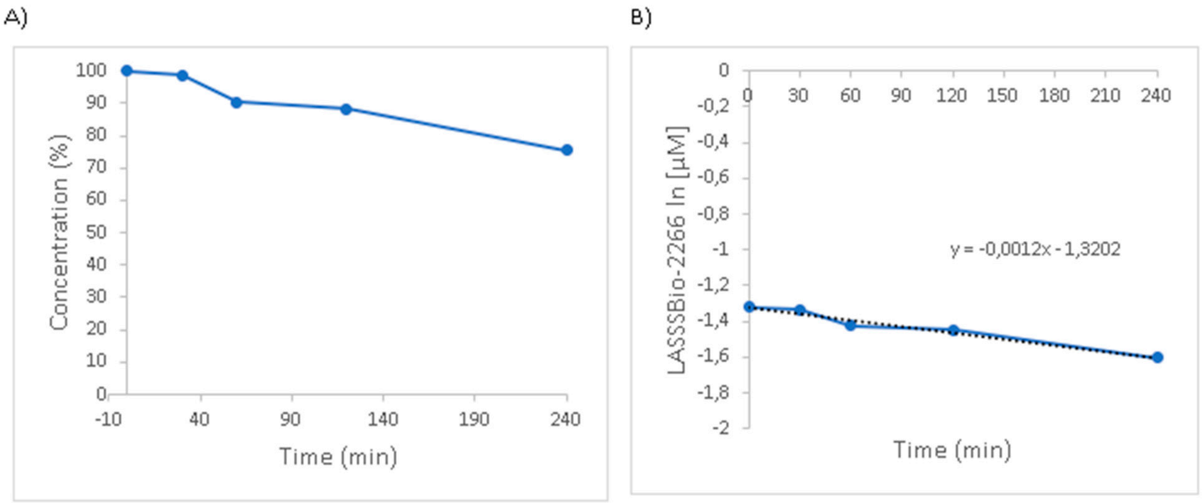


**Figure S37.** A) Plasma stability profile of (**9**); B) First order rate constant (k) for elimination.

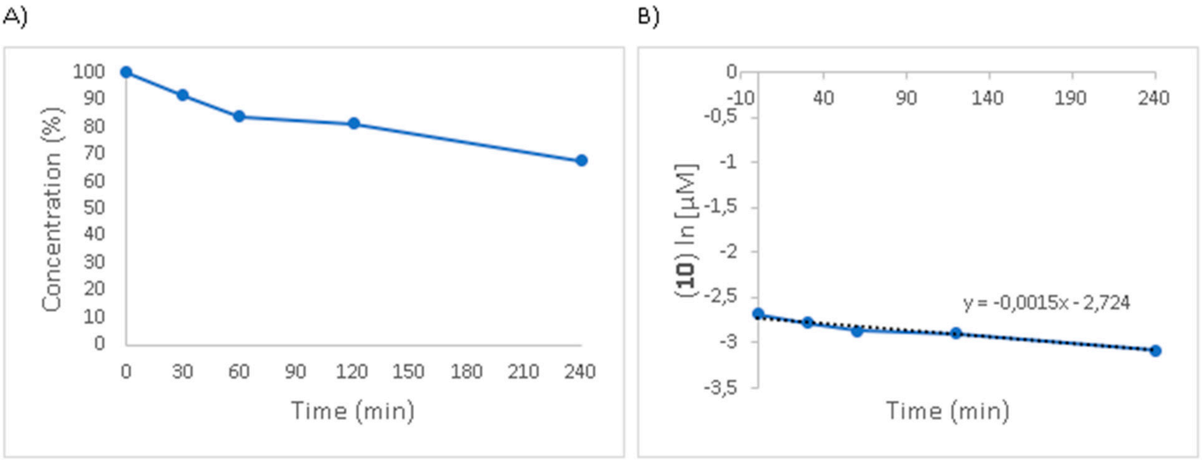


**Figure S38.** A) Plasma stability profile of (**10**); B) First order rate constant (k) for elimination.

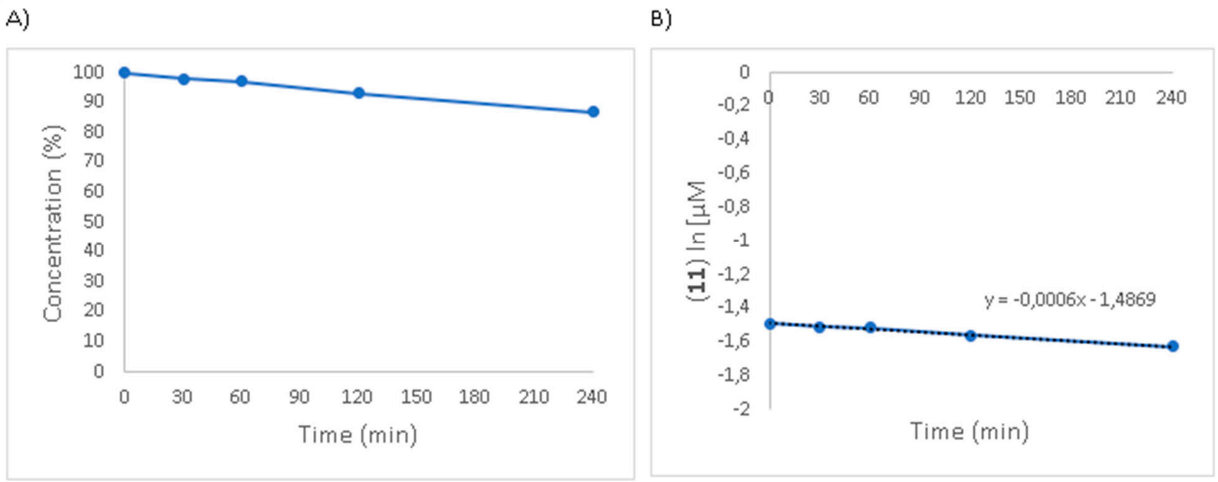


**Figure S39.** A) Plasma stability profile of (**11**); B) First order rate constant (k) for elimination.

**Table S3.** Evaluation the agonist effect against the CB1 receptors of the pyrazolamides (**7-11**).

| Compound | % of control agonist* response activity at 1 µM | % of control agonist* response activity at 10 µM |
|---|---|---|
| (**7**) | 10.4 | 25.3 |
| (**8**) | 6.5 | 14.4 |
| (**9**) | 16.9 | 23.6 |
| (**10**) | 12.5 | 26.1 |
| (**11**) | 6.7 | 4.6 |
| * The control agonist used in the evaluation of agonist activity in CB1 cannabinoid receptors was CP 55,940 ($EC_{50}$ = 3.3E-11 M) | | |

**Table S4.** Evaluation the agonist effect against the CB2 receptors of the pyrazolamides (**7-11**).

| Compound | % of control agonis* response activity at 1 µM | % of control agonist* response activity at 10 µM |
|---|---|---|
| (**7**) | 0.2 | -78.1 |
| (**8**) | -12.8 | -157.8 |
| (**9**) | -1.1 | -41.5 |
| (**10**) | -18.6 | -102.8 |
| (**11**) | -69.8 | -119.6 |
| * The control agonist used in the evaluation of agonist activity in CB2 cannabinoid receptors was WIN 55212-2 (EC50 = 2.4E-10 M) | | |

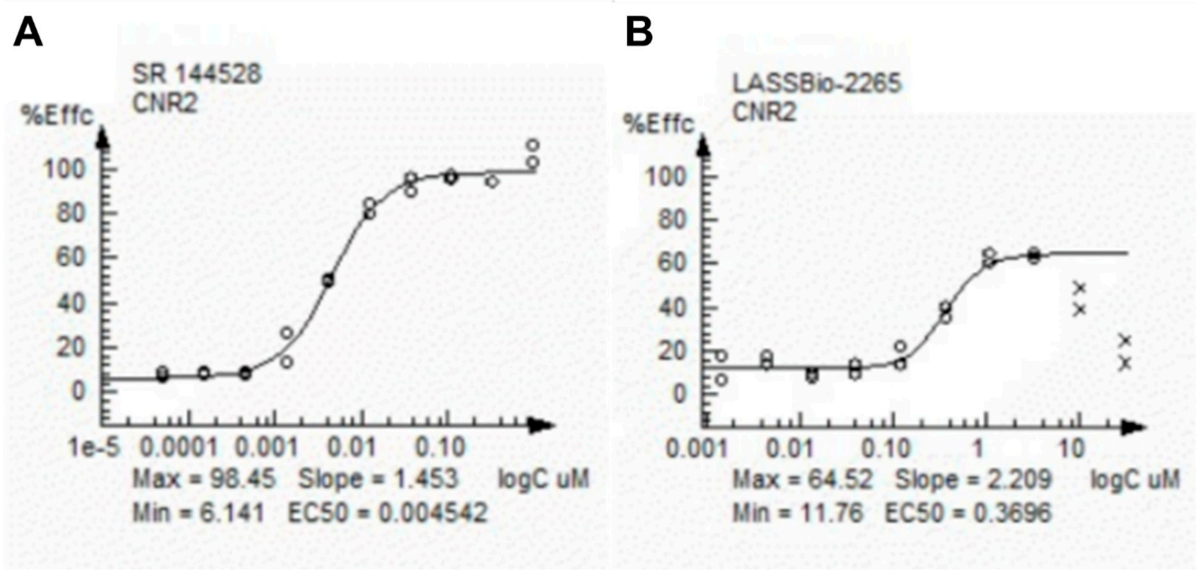


**Figure S40.** Dose-response curve of inverse agonism for cannabinoid receptor 2 (CB2) performed by the company Eurofins-CEREP: (**A**) control inverse agonist SR 144528 and (**B**) LASSBio-2265 (**11**).

**Table S5.** Raw data from the inverse agonism assay performed by the company Eurofins-CEREP

| Compound Name | Assay Format | RC50 | Unit | Hill | Curve Bottom | Curve Top | Max Response |
|---|---|---|---|---|---|---|---|
| CP55940 | Agonist | 0.001022727 | uM | 1.3517 | -5.1253 | 100 | 102.06 |
| SR 144528 | Inverse Agonist | 0.004542448 | uM | 1.4529 | 6.1415 | 98.455 | 106.31 |
| LASSBio-2265 | Inverse Agonist | 0.3695975 | uM | 2.209 | 11.761 | 64.52 | 63.063 |
